# Insights into the evolution, virulence and speciation of *Babesia MO1* and *Babesia divergens* through multiomics analyses

**DOI:** 10.1080/22221751.2024.2386136

**Published:** 2024-08-15

**Authors:** Pallavi Singh, Pratap Vydyam, Tiffany Fang, Karel Estrada, Luis Miguel Gonzalez, Ricardo Grande, Madelyn Kumar, Sakshar Chakravarty, Vincent Berry, Vincent Ranwez, Bernard Carcy, Delphine Depoix, Sergio Sánchez, Emmanuel Cornillot, Steven Abel, Loic Ciampossin, Todd Lenz, Omar Harb, Alejandro Sanchez-Flores, Estrella Montero, Karine G. Le Roch, Stefano Lonardi, Choukri Ben Mamoun

**Affiliations:** a Department of Internal Medicine, Section of Infectious Diseases, Yale School of Medicine, New Haven, CT, USA; b Unidad Universitaria de Secuenciacion Masiva y Bioinformatica, Instituto de Biotecnologia, Universidad Nacional Autonoma de Mexico, Cuernavaca, Morelos, Mexico; c Laboratorio de Referencia e Investigación en Parasitología, National Center for Microbiology, Instituto de Salud Carlos III, Majadahonda, Spain; d Department of Computer Science and Engineering, University of California, Riverside, CA, USA; e LIRMM – Université de Montpellier, CNRS, Montpellier, France; f AGAP Institut, Université de Montpellier, CIRAD, INRAE, Institut Agro, Montpellier, France; g MIVEGEC, Univ. Montpellier, CNRS, IRD, CHU, Montpellier, France; h Unité Molécules de Communication et Adaptation des Microorganismes (MCAM, UMR7245), Muséum National d’Histoire Naturelle, CNRS, Paris, France; i Laboratorio de Referencia e Investigación en Infecciones Bacterianas Transmitidas por Agua y Alimentos, National Center for Microbiology, Instituto de Salud Carlos III, Majadahonda, Spain; j Institut de Biologie Computationnelle (IBC), and Institut de Recherche en Cancérologie de Montpellier (IRCM - INSERM U1194), Institut régional du Cancer Montpellier (ICM) & Université de Montpellier, Montpellier, France; k Department of Molecular, Cell and Systems Biology, University of California, Riverside, CA, USA; l Department of Biology, University of Pennsylvania, Philadelphia, PA, USA

**Keywords:** Human babesiosis, *Babesia MO1*, *Babesia divergens*, speciation, multiomics

## Abstract

Babesiosis, caused by protozoan parasites of the genus *Babesia*, is an emerging tick-borne disease of significance for both human and animal health. *Babesia* parasites infect erythrocytes of vertebrate hosts where they develop and multiply rapidly to cause the pathological symptoms associated with the disease. The identification of new *Babesia* species underscores the ongoing risk of zoonotic pathogens capable of infecting humans, a concern amplified by anthropogenic activities and environmental changes. One such pathogen, *Babesia MO1*, previously implicated in severe cases of human babesiosis in the United States, was initially considered a subspecies of *B. divergens*, the predominant agent of human babesiosis in Europe. Here we report comparative multiomics analyses of *B. divergens* and *B. MO1* that offer insight into their biology and evolution. Our analysis shows that despite their highly similar genomic sequences, substantial genetic and genomic divergence occurred throughout their evolution resulting in major differences in gene functions, expression and regulation, replication rates and susceptibility to antiparasitic drugs. Furthermore, both pathogens have evolved distinct classes of multigene families, crucial for their pathogenicity and adaptation to specific mammalian hosts. Leveraging genomic information for *B. MO1*, *B. divergens*, and other members of the Babesiidae family within Apicomplexa provides valuable insights into the evolution, diversity, and virulence of these parasites. This knowledge serves as a critical tool in preemptively addressing the emergence and rapid transmission of more virulent strains.

## Introduction

Recent years have witnessed a significant rise in the number of tick-borne disease cases reported worldwide and an increase in the populations of ticks as well as medically important pathogens transmitted by these vectors [1,2]. This threat to public health is expected to worsen with the continued changes in the natural environment, expansion of the geographic distribution of ticks and their reservoir hosts, rapid growth of the human population, and land use changes [3]. Several tick-borne pathogens are known to cause infection in humans. Among these are *Babesia* pathogens, which infect human erythrocytes and cause human babesiosis, an emerging malaria-like illness with disease outcomes ranging from mild to severe or even fatal depending on the species, and the age and immune status of the infected individual [4].

*Babesia* species are closely related to *Plasmodium*, *Toxoplasma* and *Theileria*, the agents of human malaria, toxoplasmosis, and theileriosis, respectively [4]. They have been found in vertebrate hosts throughout the world with some species capable of infecting multiple mammals, whereas others are host specific. Most cases of human babesiosis in Europe are caused by *Babesia divergens*, predominantly among asplenic patients [5]. These infections are accompanied by high parasite burden and are often fatal. Cases of babesiosis in individuals with intact spleens have also been reported [6–9]. *Babesia divergens* also infects cattle causing “red water fever” [10]. Other human babesiosis cases in Europe have been attributed to *B. venatorum* and *B. microti* [5,11,12]. In the United States of America, cases of human babesiosis have so far been linked to at least three *Babesia* species: *Babesia microti*, which accounts for most cases reported annually; *B. duncani*, which was linked to severe babesiosis cases in Washington and California; and a *B. divergens*-like species (MO-1) reported in Missouri and Kentucky [13–15]. A previous report by Hollman and colleagues identified a parasite (NR831) that shares 99.8% sequence identity at the small subunit ribosomal RNA gene (SSU rRNA) with the MO-1 isolate [16]. The parasite was isolated from eastern cottontail rabbits (*Sylvilagus floridanus*) and *Ixodes dentatus* ticks on Nantucket Island, Massachusetts [16]. However, unlike *B. divergens*, the isolate failed to cause infection in Holstein-Friesian calves, and inoculated animals remained fully susceptible upon challenge inoculation with *B. divergens* [17].

Recently, the genome sequences of two *B*. *divergens* isolates, 1802A and Rouen 87, have been reported [18,19]. The genome of the *B. divergens* 1802A strain, isolated from cattle, was reported to be 9.58 Mb in size and to encode 4,134 genes [19]. The genome sequence of the human reference strain, *B. divergens* Rouen 87, was reported by two separate research groups with one group reporting a genome size of 8.97 Mb encoding 4,097 genes [19], and the other reporting a genome size of 10.7 Mb encoding more than 3,741 genes [18]. This latest *B. divergens* Rouen 87 genome assembly was further improved by exploiting the previous sequence data using new computational tools and assembly strategies [20], with an updated size of 9.73 Mb encoding 4,546 genes [20]. Transcriptional data and gene profiling of *B. divergens* Rouen 87 revealed insights into its invasion and lifecycle, including differentially-expressed genes, using single-cell RNA sequencing [20,21]. Unlike *B. divergens*, the biology, diversity, and virulence of *B. MO1* remain completely unknown as does the relationship between these pathogens.

In this study, we report the first complete sequence, assembly, and annotation of the genome of *B. MO1* and a comprehensive analysis of its transcription and DNA methylation during its intraerythrocytic life cycle. Additionally, we used cell biological assays and multi-omics analyses to investigate the differences between *B. MO1* and *B. divergens*. Our comparative analyses offer new insights into the evolution, diversity, and virulence of these closely related parasites.

## Results

### Comparative analysis of replication rates and genomic organization in *B. MO1* and *B. divergens* parasites

**Figure 1.**
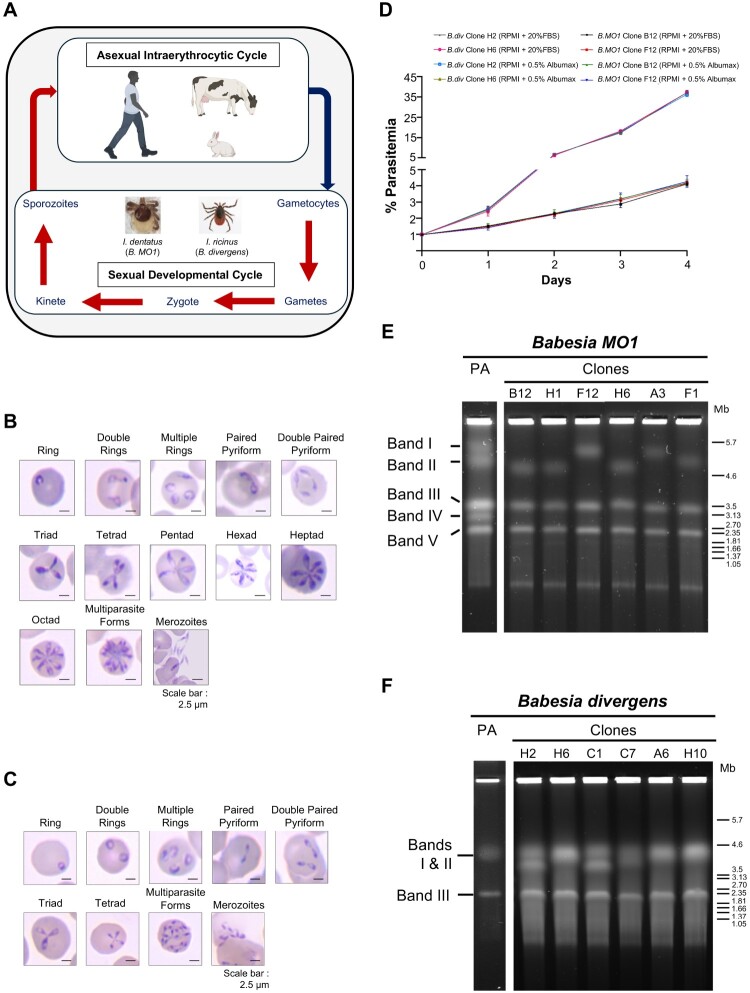
Life cycle of *B.* MO1 and *B. divergens*. A. Schematic representation of the life cycle of *B.* MO1 and *B. divergens* in vertebrate hosts (humans, cattle, cottontail rabbit) and tick vectors. B. Representative Giemsa-stained light microscopic images of the various stages of *B.* MO1 propagated in human erythrocytes in vitro. C. Representative Giemsa-stained light microscopic images of the various forms of *B. divergens* Rouen 87 grown in human erythrocytes in vitro. D. Growth of *B. divergens* Rouen 87 clones H2 and H6, and *B.* MO1 clones B12 and F12 in human RBCs in RPMI medium supplemented with 20% fetal bovine serum (FBS) or RPMI medium supplemented with 0.5% Albumax over a course of 4 days. Two independent experiments were performed in triplicates. E. Chromosomal organisation of *Babesia* MO1. PFGE shows the number and approximate sizes of bands in *B.* MO1 parental (PA) isolate: ∼5.7 Mb, ∼4.6 Mb, ∼3.5 Mb, ∼3.13 Mb and ∼2.35 Mb; the number and approximate sizes of bands in *B.* MO1 clones B12, H1, H6, and F1: ∼4.6 Mb (Chromosome I) ∼3.5 Mb (Chromosome II), and ∼2.35 Mb (Chromosome III) and *B.* MO1 clones F12 and A3: ∼5.7 Mb (Chromosome I), ∼3.5 Mb (Chromosome II), and ∼2.35 Mb (Chromosome III). The experiment was performed in biological duplicates. F. Chromosomal organisation of *B. divergens*. PFGE shows the number and approximate sizes of bands in *B. divergens* Rouen 87 parent, clones H6, A6, and H10: ∼4.3 Mb (Chromosome I and Chromosome II), and ∼2.1 Mb (Chromosome III) and *B. divergens* clones H2, C1 and C7: ∼4.3 Mb (Chromosome I), ∼4.1 Mb (Chromosome II), and ∼2.1 Mb (Chromosome III). *Hansenula wingei* and *Schizosaccharomyces pombe* DNA chromosomes were used as DNA markers. The manufacturer’s estimate of the sizes of chromosomes are indicated in Megabase pairs [13] on the right-hand side of panels E and F. The experiment was performed in biological duplicates.

*B. MO1* and *B. divergens* parasites exhibit contrasting replication rates during their intraerythrocytic life cycles. *B. MO1*, known to infect cottontail rabbits (*Sylvilagus floridanus*) and transmitted to large mammals, including humans, by *Ixodes dentatus* ticks (Figure 1A), displays an asynchronous replication rate. *B. MO1* daughter parasites divide independently, yielding a single infectious ring stage parasite that generates 2, 3, 4, 5, 6, 7, and ultimately 8 merozoites (Figure 1B). In contrast, *B. divergens* produces only four daughter parasites from each invading merozoite. However, multiple infections of a single erythrocyte by *B. divergens* merozoites often leads to the formation of more than 4 merozoites, with as many as 16 merozoites found in a single infected red blood cell (Figure 1C). To ensure the purity of clonal lines for continuous in vitro culture and subsequent multi-omics analyses, we cloned both *B. MO1* and *B. divergens* Rouen 87. Although *B. MO1* has been mostly cultured in HL-1 medium (a DMEM/F12-based medium) and *B. divergens* in RPMI-based medium, all the clones of *B. MO1* and *B. divergens* were able to grow continuously in human erythrocytes in both RPMI-based and DMEM/F12-based culture media supplemented with 20% fetal bovine serum (Figure 1D, Fig. S1). The growth of individual clones on DMEM/F12-based culture medium was indistinguishable from that on RPMI-based medium (Fig. S1). Interestingly, by measuring the growth rates of *B. MO1* clones and *B. divergens* clones, we noted significant differences. Whereas parasitemia doubled every 42–48 hours in the case of *B. MO1* clones, it doubled every 16 to 18 hours in the case of *B. divergens* clones (Figure 1D, Fig. S1).

We further examined the chromosomal organization of the nuclear genomes of *B. MO1* and *B. divergens* Rouen 87 using pulse field gel electrophoresis (PFGE) analysis. The *B. MO1* parent exhibited five bands with sizes around ∼5.7 Mb, ∼4.6 Mb, ∼3.5 Mb, ∼3.13 Mb, and ∼2.35 Mb (Figure 1E). Interestingly, clonal lines of *B. MO1* obtained following dilution cloning displayed only three bands in PFGE analysis. *B. MO1* clones F12 and A3 had bands approximately ∼5.7 Mb (Chromosome I), ∼3.5 Mb (Chromosome II), and ∼2.35 Mb (Chromosome III). *B. MO1* clones B12, H1, H6, and F1 also exhibited three bands, including one ∼4.6 Mb (Chromosome I), while the other two matched the sizes observed in clones F12 and A3 (Chromosomes II and III), with slight differences in the size of Chromosome II observed between different clones. Together these data suggest that the parent *B. MO1* strain isolated from a cottontail rabbit was a mixture of more than one clone of *B. MO1*, each carrying three nuclear chromosomes with significantly different sizes of Chromosome I, slightly different sizes of Chromosome II and mostly similar size of Chromosome III. (Figure 1E). The chromosomal profile of *B. divergens* Rouen 87 parent and clones H6, A6, and H10 revealed three bands in PFGE, with two bands overlapping, approximately ∼4.3 Mb, covering Chromosomes I and II, and another band measuring ∼2.3 Mb (Chromosome III). Similarly, *B. divergens* Rouen 87 clones H2 and C1 exhibited three band sizes of ∼4.3 Mb (Chromosome I), ∼4.1 Mb (Chromosome II), and ∼2.3 Mb (Chromosome III) (Figure 1F). The chromosomal profile of *B. MO1* differed from that of several *B. divergens* clinical isolates from France and Spain, displaying three distinct chromosomes with varying sizes across isolates, as confirmed by PFGE and Southern blot assays (Fig. S2).

### Analysis of the nuclear and organellar genomes of *B. MO1* and *B. divergens*

Sequencing, genome assembly, annotation, and assembly quality control were conducted on genomic DNA from clones F12 and B12 of *B. MO1*. Clone F12 yielded approximately 2.7 million PacBio HiFi reads with an average length of 11.5 Kb, providing approximately 2,600x coverage for the *B. MO1* genome. The assembly of clone F12 was validated using the Bionano optical map, showing strong agreement with minor exceptions at some chromosome ends (Supplementary Table I and Fig. S3A).

Our assembly identified deficiencies in covering telomeric ends, with about 0.7 Mb missing from the 5’ end and 0.5 Mb from the 3’ end of Optical Molecule 1. Molecule 2, on the other hand, is well-covered through the assembly of Chromosome II and an additional contig. Optical Molecule 3 lacks about 0.1 Mb at the 3’ end. These gaps may be due to the repetitive nature of telomeres. Interstitial telomeric repeat sequences were identified using RepeatMasker [22], including an ∼11 Kb internal transcribed spacer (ITS) [23] at the 5’ end of Chromosome II, a ∼7 Kb ITS at the 3’ end of Chromosome II, and a ∼5 Kb ITS at the 5’ end of Chromosome III (Fig. S3A). There are eleven unplaced contigs totaling ∼965 Kb, none containing an ITS. For clone B12, we obtained ∼2.8 million PacBio HiFi reads with an average read length of ∼11.9 Kb, totaling ∼33.8 billion bases, providing ∼2,800x coverage of the *B. MO1* genome (assuming a 12 Mb genome). The B12 assembly aligns well with the optical map, except for the 5’ and 3’ ends of Chromosome I and the 3’ end of Chromosome II. RepeatMasker analysis revealed ITS sequences at these ends, including an ∼9 Kb ITS at the 5’ end of Chromosome II, an ∼8 Kb ITS at the 3’ end of Chromosome II, a ∼7 Kb ITS at the 5’ end of Chromosome III, and a ∼9 Kb ITS at the 3’ end of Chromosome III (Fig. S3B). There are nine unplaced contigs totaling ∼1071 Kb, with none containing an ITS. Fig. S4 shows a synteny plot, indicating strong agreement among the F12, M12, and parental *B. MO1* assemblies, with minor differences. These include a 256 Kb insertion on Chromosome I in F12 compared to B12 and a ∼136 Kb insertion on Chromosome III in B12 compared to F12. Telomeric variations may account for differences in chromosome size between clones. For the *B. divergens* Rouen 1987 strain, we obtained ∼186,000 Oxford Nanopore Technologies (ONT) reads with an average length of ∼5.4 Kbp. The assembly, polished with Illumina reads, revealed ITS sequences at Chromosome ends. There are nine unplaced contigs totaling ∼363 Kb. Supplementary Table II summarizes key statistics of these new genome assemblies. Notably, the *B. MO1* F12, B12, and Rouen assemblies share similarities in total length (11 Mb), chromosome count, N50, GC content, and genome content completeness (Supplementary Table III). Nucleotide-level comparisons show high sequence similarity between F12 and B12 assemblies, especially in non-telomeric regions of Chromosomes I-III, with pronounced repetitive content at telomeric ends (Fig. S4). Similar patterns were observed when comparing *B. MO1* F12 to the parental strain (Fig. S4). Notably, Chromosome correspondence differs between *B. divergens* Rouen 87 and *B. MO1* F12, with significant telomeric sequence dissimilarity and a notable ∼600 Kb insertion in *B. divergens*.

Gene annotations for the *B. MO1* F12 clone were conducted using FunAnnotate (https://github.com/nextgenusfs/funannotate) and PAP (https://github.com/kjestradag/PAP) pipelines. The gene annotations for *B. divergens* Rouen 87 strain were transferred to the improved assembly using the PATT (https://github.com/kjestradag/PATT) pipeline. The gene models for *B. MO1* were established based on annotations from evolutionarily related species, and further refined using PacBio Iso-seq data specific to *B. MO1* (refer to Methods for details). These analyses yielded 4,569 gene models for *B. MO1* clone F12 and 5,274 for *B. divergens* (Supplementary Table I). The annotated genome of *B. MO1* revealed that all the enzymes of the glycolytic pathway and tricarboxylic acid cycle are present in the genome (Supplementary Tables IV and V). Our analysis also identified 20 members of GPI-anchored proteins (Supplementary Table VI) and 21 members of Apicomplexan Apetala 2 (ApiAP2) family (Supplementary Table VII).

**Figure 2.**
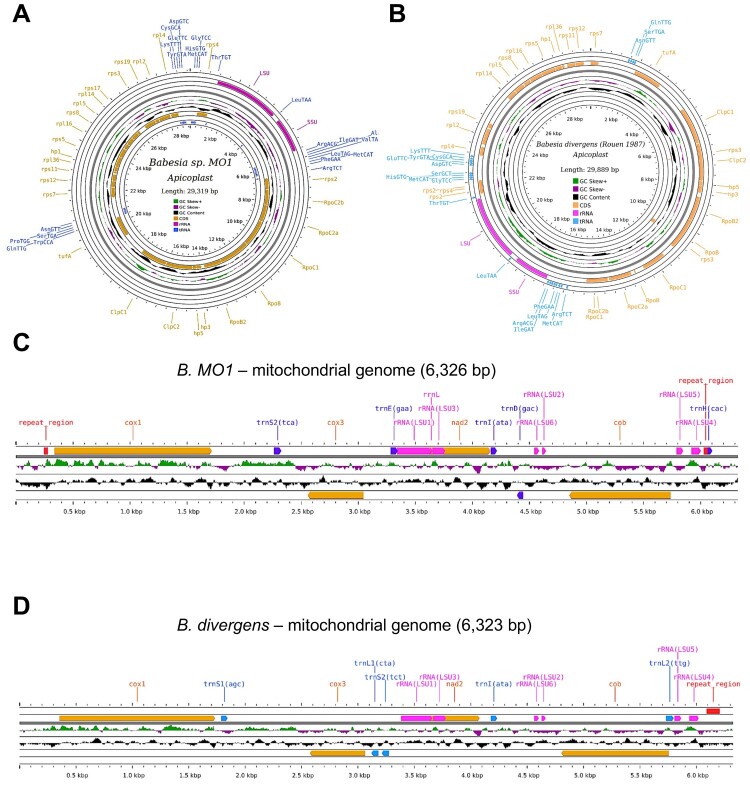
Apicoplast and mitochondrial genomes of *Babesia* MO1 and *B. divergens*. A-B. Graphical circular map of the apicoplast genome of *B.* MO1. and *B. divergens* Rouen 1987, respectively. C-D. Linear map of the mitochondrial genome of *B.* MO1 and *B. divergens* Rouen 1987, respectively. Orange arrows represent genes encoding proteins involved in the electron transport chain, including *cox1*, *cox3*, *nad2*, and *cob*. The genes encoding ribosomal RNA (rRNA) are depicted in pink colour. Different tRNA encoding genes are displayed in purple colour.

The mitochondrial and apicoplast genomes of *B. MO1* were further analyzed and compared to those of *B. divergens*. The mitochondrial genome of *B. MO1* is a linear molecule spanning 6.3 kb, while its apicoplast genome is circular, comprising 29.3 kb. The sizes of both mitochondrial and apicoplast genomes in *B. divergens* closely mirror those of *B. MO1*. The apicoplast genomes in both organisms are circular molecules measuring 29.3 kb for *B. MO1* and 29.9 kb for *B. divergens*, with A + T content of 86.4% and 86.6%, respectively. Notably, the apicoplast genome of *B. MO1* contains twenty-seven open reading frame (ORF) genes, while *B. divergens* has twenty-six. The *B. MO1* apicoplast genome includes sixteen ribosomal proteins, twenty-three tRNAs, two ribosomal RNAs (LSU and SSU), five RNA polymerases, and five additional proteins (ClpC1, ClpC2, and TufA) (Figure 2A). In contrast, the *B. divergens* apicoplast genome comprises seventeen ribosomal proteins, twenty tRNAs, two ribosomal RNAs (LSU and SSU), seven RNA polymerases, and five other proteins (ClpC1, ClpC2, hp3, hp5, and TufA). Some apicoplast-encoded transcripts in *B. divergens* are polycistronic, including rps2, rps3, RpoB, and RpoC1 (Figure 2B). The mitochondrial genomes of *B. MO*1 and *B. divergens* are characterized as monocistronic with sizes of 6326 bp and 6323 bp, respectively. Both mitochondrial genomes encode four genes (*cob, coxI, coxIII*, and *nad2*) and five tRNAs (Figure 2C and D). Additionally, the *B. MO1* mitochondrial genome codes for seven rRNAs, while the *B. divergens* mitochondrial genome codes for six rRNAs (Figure 2C and D).

#### Comparative genomic and phylogenetic studies of *B. MO1* and *B. divergens* revealed unique genetic relationships and synteny patterns

**Figure 3.**
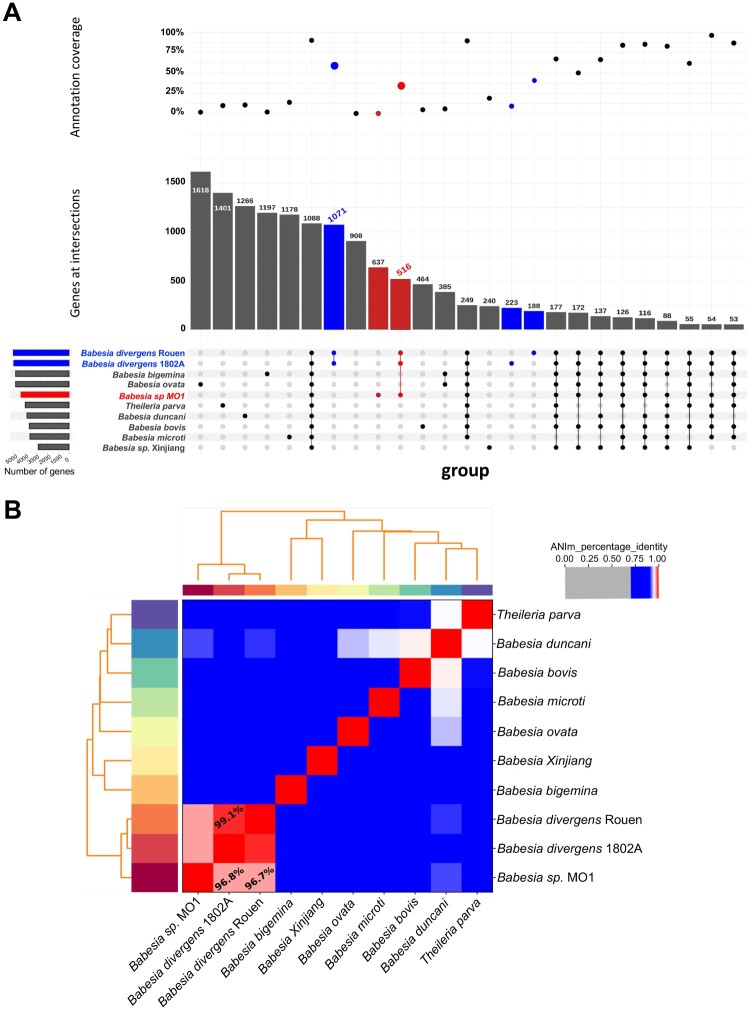
Evolutionary analysis of *Babesia* MO1 genome. A. Upset plot depicting orthogroups between *B.* MO1 and other apicomplexans. In the upper panel, the percentage of annotated proteins for shared or unique ones from a given organism is presented. In the middle panel, the total number of unique or shared proteins from a given organism is depicted. The lower panel represents the intersection or uniqueness of a given species with horizontal bars at the left side, representing the total number of genes for a given species. B. Heatmap of ANI values between *Babesia* species and *Theileria parva*. Higher values (red colour) correspond to greater nucleotide similarity between the genomes.

**Figure 4.**
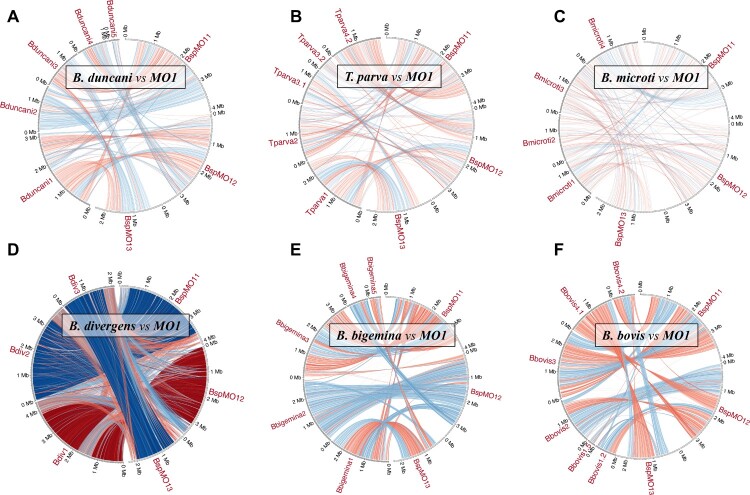
Circos synteny plots. The chromosomes of *B.* MO1 are illustrated on the right semicircle on all circular plots, and the chromosomes of the other organisms are on the left semicircle (A: *B. duncani*, B: *T. parva*, C: *B. microti*, D: *B. divergens* Rouen 87*,* E: *B. bigemina,* F: *B. bovis*); blue arcs indicate syntenies, red arcs indicate syntenies involved in a reversal; the intensity of the colour is proportional to the level of collinearity; the number after the species’ name refers to the chromosome number (when chromosomes are broken into pieces, fragments).

Genomic sequences from various Piroplasmids enabled gene comparisons among *B. divergens* Rouen 87, *B. divergens* 1802A, *B. bigemina*, *B. ovata*, *B. MO1*, *Theileria parva*, *B. duncani*, *B. bovis*, *B. microti*, and *B. sp. Xinjiang*. Our analysis found 1,088 common genes across all species, with 637 genes unique to B. *MO1*, mainly with unknown annotations. Additionally, 223 genes were unique to *B. divergens* 1802A, 188 to *B. divergens* Rouen 87, and 516 were shared among *B. divergens* 1802A, *B. divergens* Rouen 87, and *B. MO1* (Figure 3A). Genome comparisons showed that *B. divergens* 1802A and *B. divergens* Rouen 87 shared approximately 99.1% average nucleotide identity (ANI), while the ANI between *B. divergens* Rouen 87 and *B. MO1* was slightly lower at 96.7% (Figure 3B). *B. MO1* exhibited significant synteny with *B. divergens* Rouen 87, *B. bigemina*, and *B. bovis*, and lesser synteny with *B. duncani*, *T. parva*, and *B. microti* (Figure 4).

**Figure 5.**
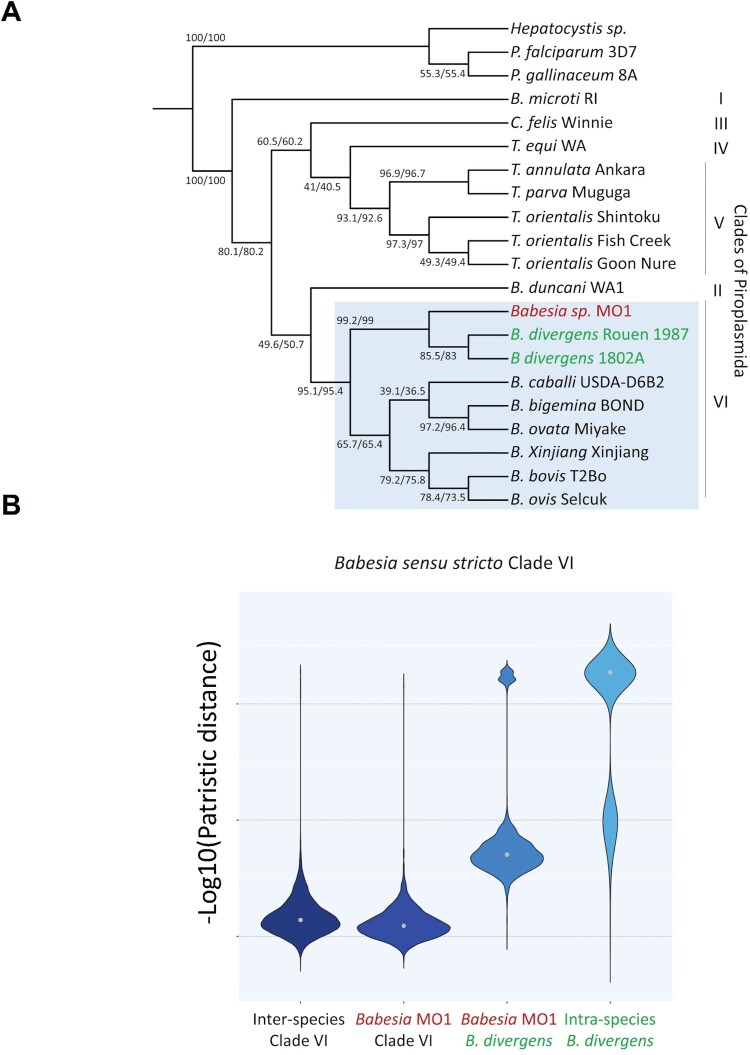
Piroplasmida species phylogeny inferred from phylogenomic analysis. A. Species phylogeny obtained by super matrix and super tree phylogenomic approaches. All bootstrap values with super matrix were at 100%. Displayed clade support values are estimated in the case of super tree methods by concordance factors from the source trees of dataset #1/dataset #2. The position of *Babesia* MO1 was analyzed in relation to the two *B. divergens* isolates (highlighted in green colour in blue box). *B.* MO1 from the present study is in red (highlighted in blue box). *Hepatocystis* sp. (ex *Piliocolobus tephrosceles* 2019), *Plasmodium falciparum* 3D7 and *P. gallinaceum* 8A were taken as outgroup. B. Summary of the genetic exchanges between Piroplasmida species based on patristic distances. A matrix of patristic distances was calculated from the 2499 trees of dataset #1 for all pairs of species. Grey dot: median of the distribution. Comparisons between species of Clade VI, between *B.* MO1 and species of Clade VI, between *B.* MO1 and two strains *B. divergens*, and between two strains *B. divergens* are shown.

Phylogenomic analysis reconstructed the evolutionary history of *B. MO1* using supermatrix and supertree methods. Two sets of orthologous genes were considered, with Dataset 1 containing ∼2500 groups and Dataset 2 including only groups with at least one outgroup sequence. The Matrix Representation with Parsimony (MRP) method generated a most parsimonious tree, with strong support for clades in Dataset 2 and significant support for most clades in Dataset 1 (Figure 5A, Fig. S6A and B). The analysis confirmed that *B. MO1* belongs to *Babesia sensu stricto* clade VI, closely related to *B. divergens* but placed outside its subclade. Confidence values provided 99% support for the *B. MO1* clade. Multiple computational approaches supported the distinct placement of *B. MO1* from *B. divergens*, indicating a close yet distinct relationship (Fig. S6C and S6D).

Patristic distances (PD) from trees in Dataset 1 characterized the speciation between *B. MO1* and *B. divergens*, showing a closer relationship in the species tree constructed through phylogenomic methods (Figure 5B). The distribution of –log10(PD) suggested recent evolution of *B. MO1* from *B. divergens*, with greater distances between *B. MO1* and *B. divergens* than between different *B. divergens* isolates. This evidence for recent speciation was reinforced by observing a shorter genetic distance between *B. MO1* and *B. divergens* compared to other *Babesia* species in Clade VI. Using PD values, approximately 75 genes were categorized into low, medium, and high groups among 22 gene ontology (GO) identities (IDs). Low-distance genes were associated with processes like protein folding, while high-distance genes were linked to mRNA maturation and degradation. This analysis also identified differences in metabolic processes, such as pyrimidine and isoprenoid biosynthesis pathways, indicating potential distinctions in cellular metabolism and adaptation to host environments between *B. MO1* and *B. divergens* (Fig. S7).

#### Regulation of gene expression, epigenetics, and chromatin structure in *B*. *MO1*

**Figure 6.**
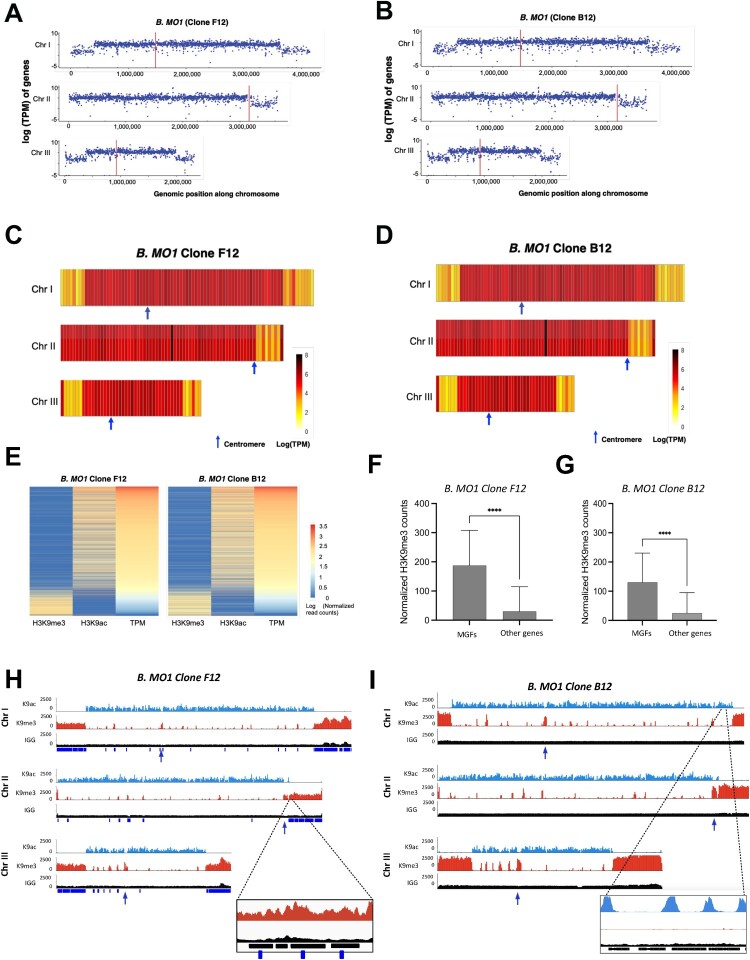
Transcriptomic profile and epigenomic landmarks of *B.* MO1. A-B. Logarithms of the TPM counts in *B.* MO1 clones F12 (panel A) and B12 (panel B) were used as expression values for each gene across the three chromosomes using the R package ggplot2. C-D. RNA-seq data of *B.* MO1 clones F12 (panel C) and B12 (panel D) as normalized heat maps across the three chromosomes. Chromosomes were divided into 50 kb bins and the average of the log TPM of genes within each bin was calculated. *n* = 2 biologically independent samples. E. Comparison between epigenetic marks and gene expression in *B.* MO1 clones F12 and B12. Heat maps were built using normalized log_2_H3K9me3 and H3K9ac read counts in addition to the RNA-seq TPM levels of each gene. Read counts for H3K9me3 and H3K9ac were normalized to millions of mapped reads and gene length, whereas TPM was determined by Stringtie. Genes were ranked from high to low TPM highlighting the correlation and anti-correlation between transcript abundance and the H3K9ac3 and H3K9me3 marks, respectively. F-G. Normalized H3K9me3 counts in multigene families, and other genes encoded by *B.* MO1 clones F12 (panel F) and B12 (panel G) (unpaired t-test with Welch’s correction, *P* < 0.0001) n = 2 biologically independent samples. H-I. Heterochromatin and euchromatin distribution across the three chromosomes of *B.* MO1 clones F12 (panel H) and B12 (panel I). Tracks correspond to H3K9ac3 ChIP [1], H3K9me3-ChIP (middle), and IgG control (bottom) and were normalized to millions of mapped reads. n = 2 biologically independent samples.

To gain further insights into the biology of *B. MO1*, RNA-seq experiments were performed for both clones. Normalized reads (Transcripts Per Million (TPM)) were plotted across the genome (Figure 6A and 6B) and binned in 50-kb windows (Figure 6C and 6D). Similar to what was observed in apicomplexan parasites that possess genes involved in antigenic variation, a significant decrease in the expression of genes belonging to MGF families localized near the telomeres was detected indicating that these genes may be repressed allowing for possible mono-allelic expression (Figure 6B and 6C) as described in *P. falciparum* [24]. Overall, RNA-seq data identified significant reads for 4540 (99.4%) of the 4569 predicted annotated *B. MO1* genes indicating that most genes are expressed during the intraerythrocytic life cycle and are potentially needed for parasite survival in the host red blood cells. Not surprisingly, the most highly expressed genes were genes involved in translation, ubiquitin proteasome system, cell cycle, ATP hydrolysis-coupled proton transport, as well as histone core proteins indicating active metabolic activity and parasite maintenance by standard housekeeping genes. Amongst the 491 genes that were found repressed with fewer than 10 TPM, nearly all did not have obvious homologues in other organisms, although many (213 of 491, 43.4%) are members of the variant erythrocyte surface antigen *vesa1, vesa2*, or identified UMGF multi-gene families*.* Interestingly, 15 of the MGF genes have over 300 TPM, placing them in the top 1000 most highly expressed genes, perhaps indicative of an antigenic variation mechanism where only a small number of them are highly expressed at any given time. Of the reads that mapped against the genome, 5.76% fall within intergenic regions and could represent long non-coding RNAs (lncRNAs) that have been shown to play a role in many biological processes including sexual differentiation [25–27] and antigenic variation [28,29].

To further examine the possible relationship between epigenetics and gene expression, we conducted chromatin immunoprecipitation assays followed by next generation sequencing (ChIP-seq) using antibodies against tri-methylated histone 3 lysine 9 (H3K9me3) and acetylated histone 3 lysine 9 (H3K9ac) as markers for heterochromatin and euchromatin marks, respectively. High Pearson correlation coefficients within each ChIP-seq pair of replicates confirm the reproducibility of our experiment (Supplementary Table VIII A and B). Negative correlation coefficients between H3K9me3 and H3K9ac samples demonstrate that, similarly to what is observed in eukaryotes including apicomplexan parasites, euchromatin and heterochromatin marks are mutually exclusive (Figure 6H and 6I). We also confirmed a large heterochromatin cluster near the telomeric and sub telomeric regions of all chromosomes surrounding multigene families. We also demonstrate that similar to what was observed in *B. duncani* [30], genes that belong to MGFs are significantly enriched in H3K9me3 marks (Figure 6F and 6G). Many of the genes annotated as hypothetical proteins and localized in telomeres ends were marked by strong histone H3K9me3 mark signal. Considering their genomic localization and their enrichment in heterochromatin marks, these genes could be involved in immune evasion. Additional histone H3K9me3 marks were also observed throughout the genome in repressed genes (Figure 6H and 6I). These genes could be involved in either immune evasion or genes expressed in the tick or involved in sexual differentiation. The euchromatic marks, on the other hand, are enriched in the promoters of active genes (Figure 6H and 6I) and their intensity correlates with transcript abundance (Figure 6E). Overall, our transcriptomic and epigenetic study further confirms that histone marks correlate with gene expression and that silencing is associated with repressed genes either involved in sexual differentiation or antigenic variation.

**Figure 7.**
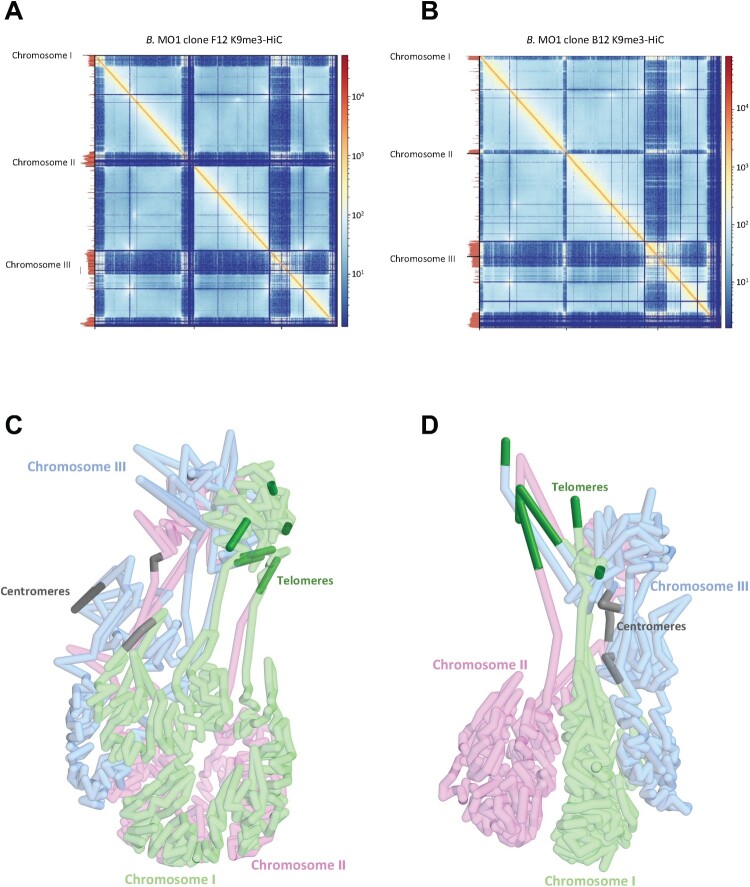
*Babesia* MO1 3D-genome. A-B. Hi-C contact maps coupled with H3K9me3 ChIP-seq tracks (left) of *B.* MO1 clones F12 and B12 (10-kb kb bins). Tracks are scaled to chromosome lengths. C-D. 3D genome structures of *B.* MO1 clones F12 and B12 derived from the contact map interactions. Chromosomes one, two, and three correspond to green, pink, and blue sections respectively. Dark green and grey represent the telomeric regions and centromeres.

The impact of MGFs on the overall chromatin organization was investigated using chromatin conformation capture (or Hi-C) on *B. MO1* clones, and intrachromosomal and interchromosomal interactions identified from HiC reads binned at 10-kb resolution. The contact maps shown in Fig. S8 indicate no major mis-assemblies in the chromosome cores, although many reads could not be mapped in the sub telomeric or highly repetitive regions, consistent with what was observed to a lesser extent in the *P. falciparum* genome [31]. When successfully mapped, sub telomeric regions or regions mapped to potential MGFs or heterochromatin marks were however detected as strongly interacting with each other confirming the formation of a possible heterochromatin cluster for most identified MGFs (Figure 7A and 7B). The acrocentric centromeres were found to interact with each other and present a distinct pattern between *B. MO1* (F12 and B12 clones) and *B. divergens* (see Figs. S8, S9, and S10). To confirm the genome-wide chromatin organization of *B. MO1* and *B. divergens*, we constructed 3D models from the Hi-C contact maps using PASTIS [32] **(**Figure 7C and 7D). In all models, the centromeres and heterochromatin/telomeres cluster together in distinct regions within the nucleus, an organization similar to what was reported in apicomplexan parasites including that of the *B. microti* and *B. duncani* genomes [30]. The strong co-localization of genes with H3K9me3 marks that included most *Babesia* MGFs confirm a tight control of *vesa* and MGF gene regions at the epigenetics and chromatin structure levels (Figure 7A and 7B).

#### Evolution of multigene families in *B. MO1* and *B. divergens*

**Figure 8.**
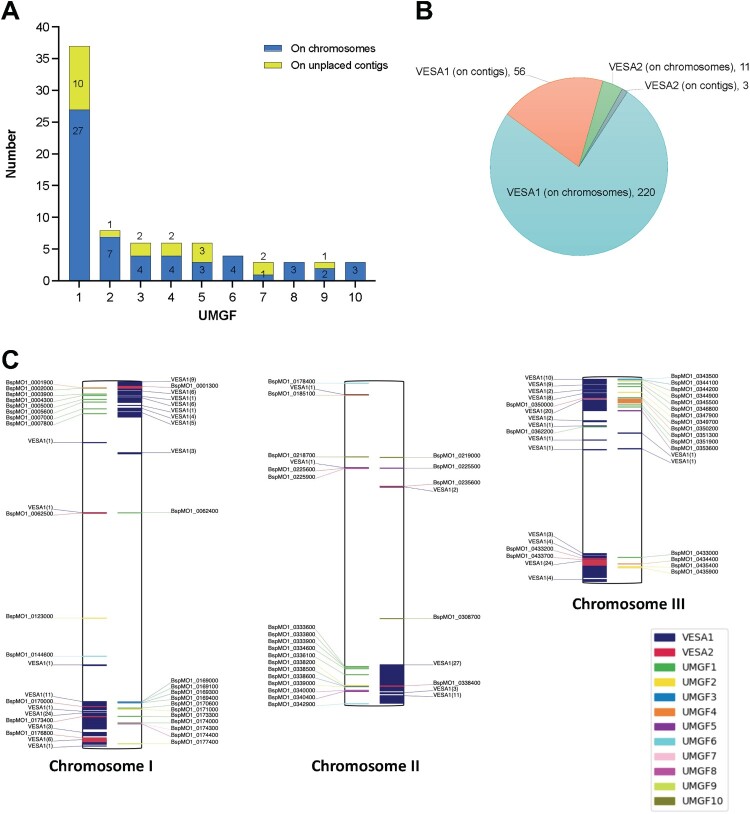
Multi-gene families of *B.* MO1 and their chromosomal localization*.* A. Plot depicting the unique multigene families (UMGFs) in *B.* MO1. The blue bars depict the genes localized on one of the three chromosomes, whereas the yellow bars denote the genes found on stray contigs. B. Distribution of *B.* MO1 *vesa*1 and *vesa*2 genes on either chromosomes or stray contigs. C. Localisation of *vesa* genes and UMGFs members on the three *B.* MO1 chromosomes (genes localized on unplaced contigs are ignored). Genes denoted on the right side of a chromosome are on the positive strand, whereas those shown on the left side are on the negative strand.

A previous study in *B. divergens* identified 359 *ves* gene encompassing three subfamilies namely, *ves1* (n = 202), *ves2a* (95), and *ves2b* (62) (Supplementary Table IX) [19]. In our reannotated genome of *B. divergens* Rouen strain, we identified only 134 *vesa* genes. Interestingly, *B. MO1* expresses 290 *vesa* genes: 276 of those had a C-terminal domain (*vesa1*) while the remaining 14 did not (*vesa2*). The *vesa* genes in *B. MO1* encode proteins with an average of 617.1 aa for *vesa1* and an average of 295.8 aa for *vesa2*. In addition to this family of genes, our analysis identified 10 novel gene families (unique multigene families; UMGFs) with at least three members. Most members of these families localize to the highly repetitive telomeric regions, the largest of which, unique multigene family (UMGF) 1, consists of 37 members, 27 of them successfully mapped to the telomeric regions of chromosomes I-III, and the remaining 10 mapped to unassembled contigs (Figure 8A, 8B). The second largest family, UMGF2, consists of 8 members, of which 7 members mapped to the telomeric regions of one of the three chromosomes; one was mapped to unassembled contigs (Figure 8A, 8B). No homologs of these proteins are found in other apicomplexan parasites, but their genome localization is reminiscent of the localization of gene families involved in antigenic variation in other parasites including the *var* genes in *P. falciparum* [31,33–35] and or the VSG in *Trypanosoma brucei*) [36]. The role of these new gene families in parasite adaptation to its mammalian host and/or vector remains to be elucidated.

#### *B. MO1* and *B. divergens* show differing susceptibility to antibabesial drugs

We also compared the susceptibility of *B. MO1* and *B. divergens* to drugs approved for babesiosis treatment: atovaquone, azithromycin, clindamycin, quinine, as well as other antifolate drugs: WR99210, and pyrimethamine. *B. MO1* exhibited approximately 2.4-fold, 1.2-fold, 1.3-fold, and 2.9-fold lower susceptibility to atovaquone, azithromycin, clindamycin, and pyrimethamine, respectively, compared to *B. divergens* Rouen 87 (Fig. S11, Supplementary Table X). In contrast, *B. MO1* showed 2.7-fold greater sensitivity to quinine and approximately 160-fold greater sensitivity to WR99210 than *B. divergens* Rouen 87. While mitochondrial-encoded *cytb* gene and nuclear-encoded genes *rpl6* and *dhfr-ts* are known drug targets, our analysis suggested that sequence conservation between *B. divergens* and *B. MO1* might not explain the differences in drug susceptibility (Fig. S12). Interestingly, RNAseq revealed significant differences in gene expression levels, especially those genes involved in folate metabolism (Supplementary Table XI), with a 10-fold difference in glutathione synthase [23] and a 12-fold difference in dihydropteroate synthase (DHPS) expression levels between the two organisms. The gene expression variations, as well as possible mechanisms of drug detoxification through increased drug efflux, may contribute to the observed differences in drug susceptibility between these pathogens.

## Discussion

The results presented in this study provide valuable insights into the biology, genomics, and epigenetics of both *B. MO1* and its close relative, *B. divergens*. These findings reveal striking differences in the replication rates, transmission dynamics, genomic characteristics, and susceptibility to antibabesial drugs between these two pathogens. The data, which substantiate the notion that these organisms are distinct but closely related pathogens, underscore the critical importance of understanding the intricacies of these parasites, particularly in the context of their evolution and the potential for zoonotic transmission to humans.

First, we found that the two organisms display major differences in replication rates and dynamics under similar experimental growth conditions. The data suggest that *B. divergens* is better adapted to human erythrocytes compared to *B. MO1*. These differences could have implications for the severity of infection and the potential for these parasites to proliferate within their respective host populations. The different transmission pathways, involving different tick vectors (*Ixodes dentatus* for *B. MO1* and *I. ricinus* for *B. divergens*) and animal reservoirs (cottontail rabbits for *B. MO1* and cattle for *B. divergens*) highlight the complex ecological interactions shaping the epidemiology of these parasites, and suggest niche specialization. Understanding these host-vector relationships and transmission cycles is crucial for devising effective control measures and assessing the risk of human infections.

Second, at the genomic level, our analysis revealed differences in chromosomal organization, both within and between *B. MO1* and *B. divergens* isolates. While the genome size and chromosome numbers are consistent between the two organisms, the patterns observed in PFGE demonstrated varying chromosome sizes, suggesting chromosomal rearrangements. Interestingly, differences between the parental isolates and clones generated from single infected erythrocytes were also observed, indicating that both *B. MO1* and *B. divergens* undergo dynamic polymorphism during their asexual development, likely the result of extensive mitotic recombination events.

Third, the genome assembly of *B. MO1* and *B. divergens*, while achieving a high-level resolution, presented challenges, especially in fully assembling repetitive telomeric ends, despite the use of long read sequencing and optical mapping technologies. This emphasizes the need for improved methods to capture and assemble repetitive genomic regions accurately. Our analysis of the genomes of *B. MO1* and *B. divergens* highlighted telomeric regions as primary source of chromosome size variation observed in PFGE, genetic variation and the location of several genomic rearrangements. Furthermore, our analysis of Average Nucleotide Identity (ANI) values and the number of orthologous proteins between *B. MO1* and *B. divergens* strains revealed further differences between *B. MO1* and *B. divergens*. Genome relatedness indices, such as ANI, offer a rapid and readily applicable means of comparing genomes to delineate species boundaries. In prokaryotes, a 95% cutoff value is well-established for grouping genomes of the same species, but ANI distribution and cutoff values for eukaryotic species delimitation have not yet been fully defined. Nevertheless, the ANI value between *B. divergens* strains (99.1%) significantly exceeds the values observed between any *B. divergens* strain and *B. MO1* (96.8% or 96.7%, respectively). Additionally, the number of orthologs shared between *B. divergens* strains (1,071 proteins) is higher than the count shared with *B. MO1* (516 proteins). The sequence divergence between *B. MO1* and *B. divergens* results in several proteins that are unique to each organism (637 proteins in *B. MO1* and 223 or 188 in *B. divergens* strains), likely tied to their specific evolution and adaptation to their respective hosts. Furthermore, our genome assemblies were crucial in exploring the evolution and function of unique proteins encoded by multigene families, such as the previously described members of the *vesa* gene family found in both *B. MO1* and *B. divergens*. However, several multigene families remain with unknown functions and need further experimental characterization to elucidate their role in each parasite. Altogether these findings highlight the genetic diversity within these parasites and offer insights into potential genetic adaptations to specific host niches.

Fourth, RNA-seq, ChIP-Seq and Hi-C analyses revealed important differences in gene expression and regulation between *B. MO1* and *B. divergens*. For example, most of the multigene families were found to be transcriptionally silent and maintained in a large heterochromatin structure, a profile similar to that of other genes involved in antigenic variation from other apicomplexan parasites. These differences in chromosomal organization were further corroborated at the epigenetics and chromatin structure levels (Figures 6 and 7), suggesting that recombination events within heterochromatin clusters may have facilitated sub telomeric variations and the potential expansion and evolution of *vesa* genes in the analyzed clones and strains. Previous research has already noted a high incidence of mutations and sub telomeric instability in highly variable genes, such as *var* genes in the human malaria parasite, *P. falciparum* [37].

Finally, we identified major differences in drug susceptibility between *B. MO1* and *B. divergens*, highlighting the necessity of considering specific variations between closely related pathogens when designing therapeutic interventions.

In conclusion, this comprehensive study significantly advances our understanding of the biology and genomics of *B. MO1* and *B. divergens*. The findings have implications for public health, emphasizing the need for tailored approaches to prevent and manage infections caused by these parasites. Future research aimed at investigating the molecular mechanisms underlying the observed differences and exploring the ecological factors influencing the epidemiology of these pathogens are warranted.

**Materials and Methods: (** *Additional methods are in Supplemental Methods* **)**

## Ethics statement

*Babesia* MO1*, B. divergens* Rouen 87 and a *B. divergens* clinical isolate from Spain were cultured using human A^+^ blood obtained from healthy volunteer donors [6]. The blood was sourced from the American red cross (US), the Interstate Blood Bank (US), or the Blood Transfusion Center (Spain), adhering to approved protocols and in compliance with the relevant institutional guidelines and regulations.

## Gene prediction and annotation of *B.* MO1 and *B. divergens*

The *Babesia* MO1 genome was processed using the gene annotation pipeline FunAnnotate v1.8.9 (https://github.com/nextgenusfs/funannotate) and PAP (https://github.com/kjestradag/PAP) pipelines. FunAnnotate was supplied with the MO1 IsoSeq isoforms computed above, along with protein sets of *B. bigemina, B. bovis, B. microti, P. falciparum, Toxoplasma gondii, T. orientalis, T. parva* and all UniProt/SwissProt protein models. Functional annotations were obtained using InterProScan v5.55-88 with default parameters. For *B. divergens* Rouen 87, gene annotations were transferred to the improved assembly presented here using the PATT pipeline (https://github.com/kjestradag/PATT). Gene models form *B.* MO1 were constructed based on annotations of evolutionarily-related species and further refined using PacBio Iso-seq data specific to *B.* MO1*.*

## Supplementary Material

Supplemental_tables.pdf

Supplementary_Methods_Clean_followed_by_supplemental_figure_legends.docx

Supplementary figures 1 to 11.pdf

## Data Availability

All datasets generated for the current study are accessible in the NCBI/SRA repository under Bioproject PRJNA1032622 (reviewer link). Specifically, the datasets include PacBio HiFi reads (SRA accession number SRR26661633), *B.* MO1 genome, RNA-Seq (SRA accession number SRR26661632), Hi-C reads (SRA accession number SRR26661630, SRR26661631), ChIP-Seq reads (SRA accession number SRR26661627, SRR26661629, SRR26661626, SRR26661628, SRR26661625).
