# Supplementary material for: Insights into the evolution, virulence and speciation of Babesia MO1 and Babesia divergens through multiomics analyses
Source: Emerg Microbes Infect. 2024 Aug 15;13(1):2386136. doi: 10.1080/22221751.2024.2386136 (PMC11370697; doi:10.1080/22221751.2024.2386136)
Supplement: Supplemental_tables.pdf [file TEMI_A_2386136_SM4002.pdf]

Supplementary Table I : Molecule size for the optical map for *B. MO1*

Optical Molecule #	Length (bp)
1	4,416,462
2	3,532,684
3	2,290,926
4	507,370
5	272,473
6	230,786
15	137,680
7	83,877
SUM	11,472,258

Supplementary Table II. Genome comparison and gene statistics

	***Babesia divergens* (Rouen-87)**	***Babesia* MO1 (F12 clone)**	**2018 *B. divergens* Rouen assembly**
Total gene models / annotated	5,274 / 3,558	4,569 / 2,795	4,546 / 3,386
Exon mean / median / mode	472.85 / 185 / 75 & 102	458.065 / 176 / 99	727 / 352 / 65
Intron mean / median / mode	605.89 / 40 / 33	421.24 / 39 / 33	328 / 105 / 36
Average exons per gene	2.65	3.14	1.68

Supplementary Table III. Assembly statistics of *Babesia divergens* Rouen and *Babesia MO1*

	***B. divergens* (Rouen 87)**	***Babesia* MO1 (clone F12)**	*Babesia* MO1 (clone B12)	**2018 *B. divergens* Rouen (assembly ASM107745v2)**
**Total length (Mb)**	10.78	11.03	10.8	9.73
**Total chromosomes**	3	3	3	Undetermined # 5 scaffolds > 1Mb
**Unplaced contigs**	7	14	9	141
**Mean contig length (Mb)**	1.07	0.787	0.899	0.069
**Longest contig (Mb)**	4.35	3.98	3.67	2.20
**N50/L50 (Mb/contigs)**	3.95 / 2	3.65 / 2	3.49 / 2	1.08 / 4
**GC content**	45	45	45	43
**BUSCO v5 (Apicomplexa lineage)**	437/446 Complete 1/446 Fragmented 8/446 Missing	434/446 Complete 2/446 Fragmented 10/446 Missing	434/446 Complete 2/446 Fragmented 10/446 Missing	437/446 Complete 1/446 Fragmented 8/446 Missing

Supplementary Table IV. Predicted enzymes of the glycolytic pathway of *B.* MO1

Glycolysis Steps	Enzyme	Gene ID	Protein Length	RNA expression level (TPM)
Glucose				
⬇ ←	Hexokinase	BspMO1_0180700.t1	539	573.231018
Glucose-6P				
⬇ ←	Phosphoglucose Isomerase	BspMO1_0354600.t1	592	416.748413
Fructose-6P				
⬇ ←	6-Phosphofructokinase	BspMO1_0305600.t1	1339	344.636505
Fructose-1,6P2				
⬇ ←	Fructose-1,6-bisphosphate aldolase	BspMO1_0017000.t1	357	875.367493
Glyceraldehyde-3P				
⬇ ←	Glyceraldehyde-3P dehydrogenase	BspMO1_0213700.t1	336	1282.513428
Glycerate-1,3P2				
⬇ ←	Phosphoglycerate Kinase	BspMO1_0381000.t1	412	745.640198
Glycerate-3P				
⬇ ←	Phosphoglycerate mutase	BspMO1_0145800.t1	248	1116.424927
Glycerate-2P				
⬇ ←	Enolase	BspMO1_0146600.t1	442	1319.670044
Phosphoenolpyruvate				
⬇ ←	Phosphoenolpyruvate carboxykinase	BspMO1_0269300.t1	546	253.2034
Pyruvate				
⬇ ←	Lactate dehydrogenase	BspMO1_0431300.t1	338	2129.362793
Lactate				

Supplementary Table V. Predicted enzymes of the TCA cycle of *B.* MO1

B. MO1 **TCA cycle**

Krebs Cycle steps	Enzyme	Predicted Gene ID	Protein Length	C/M/S	TM	RNA expression level (TPM)
Citrate						
	Aconitate Hydratase	BspMO1_0041000.t1	914	M	-	263.30365
Isocitrate						
	Isocitrate dehydrogenase	BspMO1_0098400.t1 /BspMO1_0139600.t1	519 / 455	M	-	102.51371 / 595.400269
2-oxaloglutarate						
	2-Oxoglutarate dehydrogenase	BspMO1_0276100.t1	952	C	-	251.601196
Succinyl-CoA						
	Succinyl-CoA synthetase	BspMO1_0145000.t1 /BspMO1_0407900.t1	461	M	-	324.873474
Succinate						
	Succinate dehydrogenase	BspMO1_0256000.t1 /BspMO1_0324700.t1	273<br>624	M	-	269.766235 / 139.289917
Fumarate						
	Fumarase	BspMO1_0281100.t1	468	M	-	156.482895
Malate						
	Malate dehydrogenase	BspMO1_0431300.t1	338	M	-	2129.362793
Oxaloacetate						
	Citrate synthase	BspMO1_0118500.t1 /BspMO1_0313600.t1	361 / 608	C / M	-	267.057251 / 214.3311

Supplementary Table VI. Predicted GPI-anchored proteins of *B.* MO1

		Signal P5.0	PredGPI			
GPI-AP ID	Protein ID	Score	Specificity Score	Probability	Protein length (aa)	TPM Value
BMO1GPI1	BspMO1_0016700.t1.1	0.9782	100	Highy Probable	459	102.585358
BMO1GPI2	BspMO1_0018700.t1.1	0.9763	100	Highy Probable	479	763.598877
BMO1GPI2	BspMO1_0001800.t1.1	0.9784	99.9	Highy Probable	134	10.512491
BMO1GPI4	BspMO1_0090300.t1.1	0.9243	100	Highy Probable	555	331.263947
BMO1GPI5	BspMO1_0096800.t1.1	0.6156	100	Highy Probable	619	403.405823
BMO1GPI6	BspMO1_0088800.t1.1	0.0071	99.9	Highy Probable	158	190.414551
BMO1GPI7	BspMO1_0090900.t1.1	0.751	99.9	Highy Probable	512	837.06189
BMO1GPI8	BspMO1_0119500.t1.1	0.972	99.9	Highy Probable	232	1594.880371
BMO1GPI9	BspMO1_0120200.t1.1	0.6151	99.9	Highy Probable	733	239.136703
BMO1GPI10	BspMO1_0126800.t1.1	0.0839	99.9	Highy Probable	357	258.695862
BMO1GPI11	BspMO1_0183500.t1.1	0.9153	100	Highy Probable	483	45.006592
BMO1GPI12	BspMO1_0213300.t1.1	0.7709	99.9	Highy Probable	139	528.32074
BMO1GPI13	BspMO1_0275300.t1.1	0.9899	99.9	Highy Probable	181	569.44812
BMO1GPI14	BspMO1_0277500.t1.1	0.9811	99.9	Highy Probable	579	7.428508
BMO1GPI15	BspMO1_0342900.t1.1	0.9442	100	Highy Probable	178	3517.976807
BMO1GPI16	BspMO1_0343500.t1.1	0.9454	100	Highy Probable	152	3709.012207
BMO1GPI17	BspMO1_0320700.t1.1	0.4404	99.9	Highy Probable	201	381.76651
BMO1GPI18	BspMO1_0407700.t1.1	0.0101	99.9	Highy Probable	448	186.392136
BMO1GPI19	BspMO1_0419800.t1.1	0.0032	99.9	Highy Probable	413	330.82
BMO1GPI20	BspMO1_0431600.t1.1	0.0466	99.9	Highy Probable	345	101.67

Supplementary Table VII. Predicted AP2 proteins of *B.* MO1

Gene Name	Gene ID	Protein length (aa)	RNA Expression (TPM value)	Protein MW (kDa)	Domains
BMO1-AP2-1	BspMO1_0177400.t1	629	4.00421	68.3	AP2
BMO1-AP2-2	BspMO1_0027500.t1	680	52.212086	75.8	AP2
BMO1-AP2-3	BspMO1_0036300.t1	742	384.286835	84.1	AP2, RPT1
BMO1-AP2-4	BspMO1_0063100.t1	197	315.744598	23.3	AP2
BMO1-AP2-5	BspMO1_000584-T1	74	--	8.6	AP2
BMO1-AP2-6	BspMO1_0136500.t1	375	46.351261	41.9	AP2
BMO1-AP2-7	BspMO1_0155600.t1	401	722.822876	45.3	AP2
BMO1-AP2-8	BspMO1_0164600.t1	581	179.783752	65.2	AP2
BMO1-AP2-9	BspMO1_0196700.t1	932	456.914459	104	AP2
BMO1-AP2-10	BspMO1_0206900.t1	513	178.840546	58.7	AP2
BMO1-AP2-11	BspMO1_0279000.t1	488	388.687714	55.9	AP2
BMO1-AP2-12	BspMO1_0297600.t1	475	1053.084351	53.8	AP2
BMO1-AP2-13	BspMO1_0426700.t1	691	186.523087	75.3	AP2
BMO1-AP2-14	BspMO1_0425100.t1	669	56.950947	75.6	AP2. ACDC
BMO1-AP2-15	BspMO1_0423600.t1	459	132.788696	52.2	RPAP2_Rtr1
BMO1-AP2-16	BspMO1_003360-T1	794	--	90.6	AP2
BMO1-AP2-17	BspMO1_0103400.t1	261	159.928848	29.7	AP2
BMO1-AP2-18	BspMO1_0109600.t1	214	107.731911	25.2	AP2
BMO1-AP2-19	BspMO1_0112900.t1	148	20.662474	16.8	AP2
BMO1-AP2-20	BspMO1_0138400.t1	408	84.778854	46.2	PAP2_C
BMO1-AP2-21	BspMO1_0377200.t1	541	77.83445	61.2	AP2

Supplementary Table VIII A: Genome-wide read count Pearson correlations- ChIP-Seq analysis on *B. MO1* clone F12

H3K9me3_rep1	1				
H3K9me3_rep2	0.970557561	1			
H3K9ac_rep1	-0.226618949	-0.221437881	1		
H3K9ac_rep2	-0.225918015	-0.22061916	0.993004902	1	
IgG	0.407501319	0.407465025	0.459774564	0.458195753	1
	H3K9me3_rep1	H3K9me3_rep2	H3K9ac_rep1	H3K9ac_rep2	IgG

Supplementary Table VIII B: Genome-wide read count Pearson correlations- ChIP-Seq analysis on *B. MO1* clone B12

H3K9me3_rep1	1				
H3K9me3_rep2	0.958261066	1			
H3K9ac_rep1	-0.227423493	-0.228878465	1		
H3K9ac_rep2	-0.227801951	-0.229025069	0.992019183	1	
IgG	0.333857894	0.30096601	0.133843448	0.126827877	1
	H3K9me3_rep1	H3K9me3_rep2	H3K9ac_rep1	H3K9ac_rep2	IgG

Supplementary Table IX. Multigene families in different apicomplexan parasites.

Organism	Name of multigene family	No. of members	Associated publication
P. falciparum	*var*	50-60	PMID : 16790763
P. falciparum	*stevor*	39	PMID: 21332983
P. falciparum	*rifin*	150-200	PMID: 18197962
P. falciparum	*sera*	9	PMID: 32252804
P. knowlesi	*kir*	~68	PMID: 35677565
P. vivax	*vir*	~346	PMID: 19036639
P. chabaudi	*cir*	~200	PMID: 22458863
P. cynomolgi	*cyir*	~256	PMID: 22863735
P. berghei	*bir*	~180	PMID: 26996203
P. yoelii	*yir*	~800	PMID : 12368865
B. bovis	*smorf*	44	PMID: 22138017
B. bovis	*ves*	~135	PMID:17953480
B. divergens	*vesa*<br>*ves 1α, ves1β, ves2*	134<br>(359)	This study.<br>(PMID: 24799432)
B. MO1	*vesa1*<br>*vesa2*	276<br>14	This study.
B. duncani	*Bdumgf*<br>*Bdomgf*	73<br>105	PMID: 37055610
B. microti	*bmn*	10	PMID: 22833609

Supplementary Table X. Comparison of half minimal inhibitory concentration (IC_{50}) of various antiparasitic drugs between clones of *B.* MO1 and *B. divergens* Rouen 87.

Antiparasitic drug (Target)	*B.* MO1 B12	*B.* MO1 *F12*	*B.divergens* Rouen87 Clone H2	*B.divergens* Rouen87 Clone H6	Fold difference
Atovaquone (Cyt-b)	11 ± 0.7 nM	10 ± 1.1 nM	4.5 ± 0.9 nM	4.7 ± 0.02 nM	2.4
Azithromycin (RPL6)	30 ± 2 uM	43 ± 1.7 uM	11.6 ± 0.7 uM	24.6 ± 1.7 uM	1.2
Clindamycin	113 ± 8.2 uM	81 ± 4.2 uM	11.6 ± 0.7 uM	24.6 ± 1.7 uM	1.3
Quinine	25 ± 1.4 uM	20 ± 2.6 uM	57 ± 1.9 uM	67 ± 2 uM	2.7
WR99210 (DHFR-TS)	3.1 ± 0.04 nM	0.2 ± 0.01 nM	162 ± 4.9 nM	330 ± 8.2 nM	164
Pyrimethamine(DHFR-TS)	30 ± 2.1 uM	26 ± 1.6 uM	10 ± 0.9 uM	9 ± 0.7 uM	2.9

Supplementary Table XI. RNA -seq TPM values of folate metabolism genes

Gene Name	*B.* MO1 Clone B12	*B.* MO1 Clone F12	*B. divergens* Rouen 87
**Serine hydroxymethyltransferase-1 (SHMT)**	290.896484	255.062485	108.65
**S-adenosylmethionine synthase-2 (SAMS)**	87.162872	81.135635	336.04
**Glutathione synthetase (GS)**	224.737961	232.131134	22.56
**Dihydrofolate reductase thymidylate synthase (DHFR-TS)**	297.84668	304.414795	382.95
**Adenosyl homocysteinase (AHC)**	238.004715	240.822906	328.56
**Dihydropteroate synthase (DHPS)**	214.073288	199.632187	16.59
