# Supplementary material for: Insights into the evolution, virulence and speciation of Babesia MO1 and Babesia divergens through multiomics analyses
Source: Emerg Microbes Infect. 2024 Aug 15;13(1):2386136. doi: 10.1080/22221751.2024.2386136 (PMC11370697; doi:10.1080/22221751.2024.2386136)
Supplement: Supplementary_Methods_Clean_followed_by_supplemental_figure_legends.docx [file TEMI_A_2386136_SM4001.docx]

**Supplementary Methods (Clean version)**

**Cloning of *B. MO1* isolates.** *B. MO1* in vitro culture was initiated in A^+^ human RBCs in DMEM/F12 medium at 0.5% parasitemia and 5% hematocrit (HC). The parasite culture was allowed to grow for four days and the parasitemia was measured by Giemsa-stained blood smears. The culture was subjected to serial dilution to obtain 30 parasites in 20 ml (5% HC) and 200 μl of this parasite suspension was plated per well in a 96-well plate. The culture medium of the cloning plate was replaced with fresh medium every 3^rd^ day for 21 days. On day 22, SYBR Green-I assay was performed to determine the parasite positive wells of the cloning plate. Briefly, 25 μl of culture per well from the cloning plate was transferred to a black bottom 96-well plate (Stellar Scientific, IP-DP35F-96-BLK) and mixed with 25 μl of SYBR Green-I lysis buffer (20 mM Tris, pH 7.4, 5 mM EDTA, 0.008% saponin, 0.08% Triton X-100 and 1X SYBR Green-I (Molecular Probes, 10,000X solution in DMSO, Eugene, OR, USA)) and incubated for 30 min in dark at 37°C. In addition, uninfected human RBCs (5% HC, 25 μl volume) were used as a negative control. Following the incubation, the SYBR Green-I measurement was performed on BioTek Synergy MX fluorescence plate reader with an excitation of 497 nm and emission of 520 nm. The readings from uninfected human RBCs were used as background and subtracted from the readings of the cloning plate wells in order to determine wells positive for parasites (higher SYBR Green-I readings in comparison to the negative control). Following identification of parasite positive wells using SYBR Green-I assay, the same wells were used to prepare smears for Giemsa staining and presence of parasites was confirmed using light microscopy. Six clones from parasite positive the 96-well plate were picked and expanded to 1 ml cultures and allowed to grow to 2% parasitemia before expanding them to 5 ml cultures. Two of the six clones (*B. MO1* clone B12 and clone F12) were used in this study. All the clones of *B. MO1* and *B. divergens* were subsequently also maintained and propagated in RPMI + 20% FBS and RPMI + 0.5% Albumax II for 3-4 generations before comparing their growth rates.

**Continuous *in vitro*culture of *B. MO1 and B. divergens* in human red blood cells.** *B. MO1* were obtained from BEI Resources (BEI Resources, NR-50441) and *B. divergens* parasites were a kind gift from Dr. Laura Kirkman (Weill Cornell Medicine). *B. MO1* and *B. divergens* parasites were initially cultured for 3-4 generations (replicative cycles) in DMEM/F12 + 20% heat inactivated fetal bovine serum (FBS) (Sigma, F4135) and then used for dilution cloning to isolate pure clonal lines. The clones of *B. MO1* and *B. divergens* were cultured for 6-8 generations (replicative cycles) in DMEM/F12 + 20% FBS and then shifted to RPMI 1640 (Gibco-Life Technology, 11875093) + 20% FBS and RPMI 1640 + 0.5% Albumax II (Gibco, 11021037). Subsequently, the clones of *B. MO1* and *B. divergens* were propagated in RPMI medium supplemented with 20% FBS or RPMI 1640 supplemented with 0.5% Albumax II, 1X from 50X HT Media Supplement Hybrid-MaxTM (Sigma, H0137), 1x from 200 mM L-Glutamine (Gibco, 25030-081), 1x from 100X Penicillin/Streptomycin (Gibco, 15240-062) and 1x from 10 mg/mL Gentamicin (Gibco, 15710-072) in 5% hematocrit A^+^ RBCs. The parasite cultures were maintained at 37°C under a 2% O_2_ / 5% CO_2_ / 93% N_2_ atmosphere in a humidified chamber. Culture medium was changed every 24 h, and parasitaemias were monitored by examining Giemsa-stained blood smears using a light microscope at 100x.

**DNA preparation for Oxford Nanopore and Illumina sequencing for *B. divergens* Rouen 87.** Genomic DNA (gDNA) was isolated from asynchronous *B. divergens* *in vitro* cultures with 40% of parasitemia. The gDNA was prepared using pellets of infected RBCs. Pellets were lysed with 0.15% Saponin (Sigma-Aldrich) for 30 minutes and centrifuge at 2000 x g and 4ºC for 10 minutes. The final pellets were incubated in lysis buffer (0.1 M NaCl, 50 mM Tris-HCl, pH 7.5, 1 mM EDTA, sodium dodecyl sulfate [SDS; 0.5% by volume], and 100 μg ml^-1^ of proteinase K (Sigma-Aldrich) for 16 h at 56°C. Nucleic acid was recovered by phenol-chloroform extraction, followed by ethanol precipitation. RNA was removed by RNase digestion (Roche Diagnostic GmbH, Germany) and DNA was subjected to a further round of phenol-chloroform extraction and ethanol precipitation.

**DNA preparation for Bionano Optical Map for *B. MO1*.** *B. MO1* was cultured in vitro in human RBCs to attain a parasitaemia of 8-10% at 5% haematocrit (total 100 ml). The parasite pellet was generated by centrifuging the cultures at 500 x g and used to isolate ultra-high molecular weight (HMW) genomic DNA for use in genomic optical mapping (Histogenetics) using the Bionano Prep Blood and Cell Culture DNA Isolation kit (Bionano Genomics, 80004). The DNA was quantified using Qubit dsDNA BR Assay kit. Around 0.8g of HMW DNA was labelled using the Bionano Prep direct label and stain method (Bionano Genomics, 80005) and loaded onto a flow cell to run on the Saphyr optical mapping system (Bionano Genomics). Around 1.2 Gb of data were generated per run. Raw optical mapping of molecules in the form of BNX files were run through a preliminary bioinformatics pipeline that filtered out molecules less than 150 kb in size and less than 9 motifs per molecule to generate a *de novo* assembly of the genome maps.

**Genome Sequencing and Assembly of *B. MO1* isolate F12 and B12.** DNA for clones F12 and B12 were sequenced at the Yale Center for Genome Analysis using PacBio HiFi (CCS). HiFi reads for clone F12 totaled 31.2 B bases, which translated to a ~2600x coverage of the *B. MO1* genome (assuming a genome of 12Mb). Given the abundance of sequencing data, hifiasm v0.19.6 [2] and HiCanu v.2.2 [3] were tested on (1) the entire 2600x-coverage data, (2) the 250 thousand longest HiFi reads (511x coverage, average read length = 21,043 bp), and (3) the 100 thousand longest HiFi reads (228x coverage, average read length = 27,362 bp). These six assemblies were aligned to the Bionano optical map using Bionano RefAligner Solve v3.7 to detect possible mis-joins. Based on assembly statistics, comparison with the optical map and BUSCO completeness, it was determined that the best assembly of clone F12 was obtained using hifiasm on the 100 thousand longest HiFi reads. This assembly was used as the reference *B. MO1* genome in the rest of this study. HiFi reads for clone B12 totaled 33.8 B bases, which translated to a ~2800x coverage of the *B. MO1* genome (assuming a genome of 12Mb). The same assembly strategy used for F12 was used for clone B12. The best assembly of clone B12 was obtained again using hifiasm on the 100 thousand longest HiFi reads (about 200x coverage, average read length = 24,041 bp).

**Genome Sequencing and Assembly of *B. divergens* Rouen 87 .**  DNA from a *B. divergens* culture was used for Oxford Nanopore sequencing. Sequencing libraries were prepared using the SQK-LSK109 kit with a 1µg of DNA input following the vendor’s protocol. Sequencing was performed using a MinION flow cell (v9.4). The base-calling was carried out using the software Guppy v4.0.14 with default parameters and a high accuracy error model (dna_r9.4.1_450bps_hac.cfg). A *de novo* assembly was performed using Oxford Nanopore long reads and Canu v.1.9 [3] assembler with default parameters. This assembly was corrected using Illumina reads from the already previous *B. divergens* assembly [4] and three iterations of Pilon v.1.23 [5].

**PacBio IsoSeq processing.** PacBio IsoSeq data was mapped to the *B. MO1* genome using Minimap2 with options ‘splice:hq -uf –secondary=no -C5’. The resulting alignments were fed into the PacBio cDNA_Cupcake pipeline (https://github.com/Magdoll/cDNA_Cupcake) using the script ‘collapse_isoforms_by_sam.py’ to obtain non-redundant transcript isoforms. The isoform sequences were used in the gene finding pipeline below.

**Phylogenetic and phylogenomics analyses.** Phylogenomic analysis was conducted using protein sequences from PiroplasmaDB plus *Babesia* sp. MO1 and *B. divergens* Rouen 87 genome annotation from present study, *B. duncani* WA1 [6] and three outgroup genomes, namely *Hepatocystis* sp. (ex *Piliocolobus tephrosceles* 2019), *Plasmodium falciparum* (strain 3D7) and *P. gallinaceum* (strain 8A) from PlasmoDB.

Protein sequences were compared by selecting OrthoMCL groups (supplementary method). Dataset #1 contains the 2,499 orthologous groups having a unique gene per isolate and at least four sequences. Dataset #2 contains the 1,361 orthologous groups from Dataset #1. Each group of orthologous sequences was aligned using the following procedure. First, the orthologous sequences were aligned using Muscle v5.1 [7], with default parameters. Second, the resulting alignment was filtered using HMMCleaner v1.8 [8], with default parameters. Finally, gap-only sequences and gap-only sites were removed using the splitAlignment subprogram of MACSE v2.07 [9]. For each filtered alignment of an orthologous group, we inferred a gene tree by the maximum likelihood criterion using IQ-TREE [10-13] (details given in supplemental material). PhySIC_IST and SuperTriplets require rooted trees, thus we rooted the gene trees by resorting to the outgroup method (see supplemental material for more details). We used three different supertree methods, namely MRP [14], PhySIC_IST [15] and SuperTriplets [16]. The two later require rooted trees as input, thus could only be run on Dataset #2, while MRP could analyze both datasets #1 and #2 (see supplementary data for more details).

A supermatrix analysis was carried out, both on Dataset #1 and #2 by concatenating all alignments of the orthologous groups composing a dataset. We thus obtained a supermatrix of 1,109,333 characters x 21 taxa containing only 34% of missing data for Dataset #1 and 541,931 characters x 18 taxa with 18% missing data for Dataset #2. We then estimated the most likely species tree according to each of these matrices separately, thanks to the IQ-TREE version 2 software. We used the edge-linked partition model to analyze the supermatrix [10,11], allowing each gene family to have its own evolutionary rate though all families shared the same branch lengths. We obtained branch support with the ultrafast bootstrap [12] by resampling partitions then sites within partitions [17,18].

**In vitro growth rate determination of *B.* MO1 clones and *B. divergens* clones in different culture media.** In vitro cultures of the *B.* MO1 clones B12 and F12 and *B. divergens* Rouen 87 clones H2, and H6 were initiated at 1% parasitemia in human RBCs at 5% hematocrit and sustained in RPMI medium + 20% FBS or RPMI medium + 0.5% Albumax. The parasite cultures in the aforementioned media were maintained for four days without subculturing. The respective culture media was replaced daily, and parasite growth was monitored after every 24 h by examination of Giemsa-stained blood smears using a light microscope.

**RNA-seq processing for gene-expression analysis.** RNA-seq data were assessed for quality using FastQC v0.11.8. Adapter sequences as well as the first 11 bp of each read were trimmed using Trimmomatic v0.39. Tails of reads were trimmed using Sickle with a Phred base quality threshold of 25, and reads shorter than 18 bp were removed. Reads were then aligned to the *B.* MO1 F12 genome assembly using HISAT2 v2.2.1. Only properly paired reads were retained, with filtering done using Samtools v1.11. Non—uniquely mapped reads were retained due to highly repetitive regions. PCR duplicates were removed with PicardTools MarkDuplicates v2.18.0 (Broad Institute). StringTie v2.2.1 was run with the -e parameter to estimate the abundance of each gene in TPM (transcripts per million).

**Comparative genomics.** Comparative genomics between different species of *Babesia* was performed by running OrthoMCL on the genome data obtained from PiroplasmaDB and PlasmoDB release 58. *Babesia bigemina* strain BOND*, Babesia bovis* T2Bo, *Babesia divergens* strain 1802A, *Babesia duncani* strain WA1, *Babesia microti* strain RI, *Babesia ovata* strain Miyake, *Babesia* *sp.* *Xinjiang Xinjiang*, and *Theileria parva* strain Muguga genomes were used in this analysis. OrthoMCL was run on these eight species, as well as the newly assembled genomes of *B. divergens* Rouen 87 and *B.* MO1*.* The UpSet plot was generated using R.

The pairwise comparisons between the genome of *Babesia* species was performed. First, the synteny between assemblies using the web server Genies (http://dgenies.toulouse.inra.fr/) with the Minimap2 aligner was calculated. The average nucleotide identity (ANI) between all genome pairs was calculated with PyANI v.0.2.10 (https://github.com/widdowquinn/pyani).

Synteny circos plots in **Fig 3** were obtained using mummer2circos v1.4.2 (https://github.com/ metagenlab/mummer2circos) that uses the promer algorithm in conjunction with CIRCOS.

CIRCOS plots in **Fig 4** were generated using the circoletto.pl script v.07.09.16 that uses CIRCOS v2.43.0 underneath. The used options for circoletto were: --out_size 2000 --e_value 1e-3 --untangling_off (<https://github.com/infspiredBAT/Circoletto>). We obtained orthologous proteins between *B. divergens* Rouen and B. MO1, *B. microti* and *B. bovis*, we used the ProteinOrtho v.6.0.24 software using default parameters and proteins from each genome. The genome from the mitochondrion and apicoplast organelles for *Babesia divergens* Rouen and *B.* MO1 were compared against other species (*B. ovata, B. microti, B. bovis* and *B. bigemina*) by performing a multiple alignment with MAFFT v.7.453 with the following parameters: --reorder --maxiterate 1000 --threadit 0 --retree 1. A phylogenetic tree was generated with a maximum likelihood approach by using first jmodeltest-2.1.10 to select the best tree model and then PhyML version 3.3.3:3.3.20190909-1 to generate the tree.

Gene localization plots in **Fig 5** were produced using our tool GFViewer (<https://github.com/sakshar/gene-localization-tool>).
GC-skew plots in Fig S13 were obtained using SkewIT (<https://jenniferlu717.shinyapps.io/SkewIT/>) [19]

**ChIP-seq sample preparation**

Approximately 20 million *B.* MO1 parasites per sample/per condition were pelleted and crosslinked with formaldehyde, then quenched with glycerine, and followed by a series of washes with PBS. The resulting pellet was resuspended in 1mL nuclear extraction buffer (10 mM HEPES, 10 mM KCl, 0.1 mM EDTA, 0.1 mM EGTA, 1 mM DTT, 0.5 mM AEBSF, 1X Roche protease inhibitor, 1X Roche phosphatase inhibitor) followed by a 30 min incubation on ice. 10% Igepal CA-630 was added to each sample, homogenized by passing through a 26G × 1⁄2 needle and centrifuged at 5,000 rpm to obtain the nuclear pellet. The nuclear pellets were resuspended in shearing buffer (0.1% SDS, 1 mM EDTA, 10 mM Tris-HCl pH 7.5, 1X Roche protease inhibitor, and 1X Roche phosphatase inhibitor) and transferred into 130uL Covaris tubes (PN 520045). Samples were then sonicated using a Covaris S220 (under following settings: 5 min, duty cycle 5%, intensity 140 W, 200 cycles/burst, 6°C) before adding equal volumes of ChIP dilution buffer (30 mM Tris-HCl pH 8, 3 mM EDTA, 0.1% SDS, 30 mM NaCl, 1.8% Triton X-100, 1X protease inhibitor, 1X phosphatase inhibitor). Samples were centrifuged at 13,000 rpm for 10 min at 4°C. For each sample, 13 μL protein A agarose/salmon sperm DNA beads were washed 3 times with ChIP dilution buffer without inhibitors. The washed beads were added to the diluted chromatin for 1 hr at 4°C with agitation to pre-clear the samples. ~10% of each sample by volume was set aside as input; to the remaining, 2μL of antibodies anti-H3K9me3 (Abcam ab8898), anti-H3K9ac (Diagenode C15410004), or IgG(Abcam ab46540) were added for overnight rotation at 4°C. To each sample, 25 μl of washed protein A agarose/salmon sperm DNA beads with ChIP buffer were blocked with 1 mg/ml BSA for 1 hr at 4°C, re-washed, and added to each sample for 1 hr rotation at 4°C. The bead/antibody/protein complexes were washed a total of 8 times 15 min intervals per wash): twice with low salt buffer (1% SDS,1% Triton X-100, 2 mM EDTA, 20 mM Tris-HCl pH 8, 150 mM NaCl), twice with high salt buffer (1% SDS,1% Triton X-100, 2 mM EDTA, 20 mM Tris-HCl pH 8, 500 mM NaCl), twice with LiCl buffer (0.25 M LiCl , 1% NP-40, 1% Na-deoxycholate,1 mM EDTA , 10 mM Tris-HCl, pH 8.1 ), and twice with TE (10 mM Tris-HCl pH 8, 1 mM EDTA) buffer. DNA was then eluted from the beads with two 250 μl washes of elution buffer (1% SDS, 0.1 M sodium bicarbonate) and added NaCl (55ul of 5M) to reverse crosslink overnight at 45°C. RNAse A (15 μl of 20 mg/mL) and proteinase K (2 μl 20 mg/mL) were subsequently added to the samples, incubated at 37°C and 45°C, respectively, followed by a DNA extraction via phenol/chloroform and ethanol precipitation. After precipitation, the samples were centrifuged at 13,000 rpm for 30 min at 4°C, forming pelleted DNA, washed with 80% ethanol, re-pelleted, and resuspended the DNA in 50 μl nuclease-free water. The DNA was purified with AMPure XP beads and prepared Illumina sequencing libraries using a KAPA Hyperprep kit (KK8504), followed by the NovaSeq 6000 sequencing platform (Illumina).

**ChIP-seq Analysis.** Read quality was analysed using FastQC (https://www.bioinfor- matics.babraham.ac.uk/projects/fastqc/) and trimmed adapters and low-quality bases using Trimmomatic (http://www.usadellab.org/cms/?page=trimmomatic) and Sickle (https://github.com/najoshi/ sickle). Reads were mapped against the *B.* MO1 F12 and B12 assemblies using Bowtie2 v2.4.4 (https://doi.org/10.1038/s41564-023-01360-8) while keeping non-uniquely mapped fragments and retained only correctly paired reads using Samtools (v1.11) (http://samtools.sourceforge.net). PCR duplicates were removed with PicardTools MarkDuplicates v2.18.0 (Broad Institute). To obtain per nucleotide coverage and generate browser tracks, we used BedTools v2.27.1 and custom scripts, normalizing counts by millions of mapped reads. Chromosome tracks were viewed using IGV (Broad Institute). To compare H3K9me3 levels between MGF genes and other genes, read counts for H3K9me3 (and IgG control) were calculated within each gene body using bedtools multicov. Counts were normalized to millions of mapped reads per library and gene length in kb. The background signal from the IgG control was subtracted from H3K9me3 counts, setting negative values to 0. H3K9ac read counts were also generated by bedtools multicov but including 300 bp upstream of genes as acetylation is often in promoter regions. Heatmaps were generated using normalized H3K9me3, H3K9ac, and RNA-seq TPM counts for each gene to compare histone modifications with gene expression, sorting genes by TPM. The heatmap used log-scaled counts and sorted genes from high to low TPM.

**Phylogenetic analyses.** To infer the species phylogeny, a phylogenomic analysis was conducted using protein sequences from PiroplasmaDB plus *Babesia sp.* MO1 and *B. divergens* Rouen 1987 genome annotation from present study, *B. duncani* WA1 [6] and three outgroup genomes, namely *Hepatocystis s*p. (ex *Piliocolobus tephrosceles* 2019), *Plasmodium falciparum* (strain 3D7) and *P. gallinaceum* (strain 8A) from PlasmoDB. Pseudogenes and genes encoding peptides below 100 amino acids were removed. CH-HIT was used to removed duplicated genes with following for loop for f in *.fasta; do b=$(basename $f .fasta); ../../BABESIA_2022/soft/CDHIT/cd-hit-v4.8.1-2019-0228/cd-hit -i $f -o ../CDHIT_results/${b}_noDup.fasta -c 1.00 -t 1 > ../CDHIT_results/${b}_noDup.log;done

For the analysis of orthology groups, *B. sp.* MO1*, B. divergens* Rouen 1987 and *B. duncani* genes were assigned to OrthoMCL (https://OrthoMCL.org) groups using the orthology assignment tool available through the VEuPathDB (https://VEuPathDB.org) Galaxy workspace. Proteins in FASTA format were assigned to groups based on the OG6r15 BLAST database using the default settings. Output files generated by the OrthoMCL pipeline included a mapping file between gene IDs and OrthoMCL v.6 group IDs. VEuPathDB resources including PlasmoDB.org and PiroplasmaDB.org provided OrthoMCL v.6 group IDs. A matrix containing the number of genes per OrthoMCL group was generated with a custom R script.

Protein sequences were compared by selecting OrthoMCL groups. Each group of orthologous sequences was aligned using the following procedure. First, the orthologous sequences were aligned using Muscle v5.1 [7], with default parameters. Second, the resulting alignment was filtered using HMMCleaner v1.8 [8], with default parameters. Finally, gap-only sequences and gap-only sites were removed using the splitAlignment subprogram of MACSE v2.07 [9].

For data set generation, we selected only a subset of these alignments for phylogenomic analysis. Indeed, inferring a species tree from families containing both orthologous and paralogous sequences is error prone. Thus, we only considered families with at most one sequence per taxa, maximizing the probability to consider only orthologous sequences. We restricted ourselves to gene families spanning at least four taxa (there is only one possible unrooted tree topology for three taxa). Phylogenomic inference was done using supermatrix and supertree methods. Some supertree methods require rooted trees as input. Overall, we considered two datasets: Dataset #1 contains the 2,499 orthologous groups having a unique gene per isolate and at least four sequences. Dataset #2 contains the 1,361 orthologous groups from Dataset #1 that additionally contained at least one outgroup sequence and such that the outgroup sequences were monophyletic in the corresponding gene tree (when several outgroup sequences were present).

A tree showing has been inferred by maximum likelihood through the IQ-TREE version 2 software for each gene family with the command:

iqtree2 -s OG6_100089_filtered.aln --seqtype AA -b 100 -mset LG,WAG,JTT,Blosum62 -cmax 4 --prefix OG6_100089_iqtree --quiet

where OG6_100089 is the gene family considered here.

The matrices of patristic distances (distance from one leaf to another in a phylogeny) was calculated for our 2499 trees with the following command:

for c in $(cat ../cog.list); do nw_distance -n -m m ALIPHY_DETAILS/__${c}/${c}_iqtree.treefile > patristiDistances/${c}.pdist; done

The maximum likelihood inference detailed above gave unrooted gene trees. We rooted each of them by placing the root node on the branch separating the outgroup taxa from the other ones. The outgroups in this analysis are *Hepatocystis* sp., *Plasmodium falciparum* 3D7 and *P. gallinaceum* 8A. When a gene family contained no outgroup, it could not be rooted.

The rooting was performed by the version 0.1.3 of the bpp-reroot utility from Bio++ (Dutheil et al 2006). For instance, for the OG6r15_117499 orthologous group we used the following command:

./bppReRoot input.list.file=OG6r15_117499_iqtree.treefile outgroups.file=outgroup.txt output.trees.file= OG6r15_117499.bppReRoot.nwk print.option=false

Graphic representation was performed using ggplot2 in R.

PhySIC_IST and SuperTriplet require rooted trees, thus we rooted the gene trees by resorting to the outgroup method (see supplemental material for more details). Here outgroup taxa are the two *Plasmodium* isolates together with *Hepatocystis* sp. sequences.

We inferred a piroplasma phylogeny from datasets #1 and #2. We performed both a supermatrix and a supertree analysis. We used three different supertree methods: MRP [14], PhySIC_IST [15] and SuperTriplets [16]. The two latter require rooted trees as input, thus could only be run on Dataset #2, while MRP could analyze both datasets #1 and #2.

The analysis with MRP method was conducted by using the BuM program, available online at <http://nuvem.ufabc.edu.br/bum>. We obtained a binary character matrix encoding the source trees for datasets #1 and #2 separately after trimming all branch lengths and clade support values according to the program manual. For both datasets we produced a most parsimonious tree for the character matrix by the TnT software. The analyzing script asked TnT to perform an exact search of the most parsimonious tree, which is feasible for such a small number of taxa. Below is the precise script used for analyzing Dataset #1:

log ds1_optimal.log;

mxram 1000;

nstates NOGAPS;

taxname=;

p ds1_treefiles_topo.ss;

hold 1000;

ienum;

export - ds1_optimal_MRP.tre;

quit;

The computations on datasets #1 and #2 ended up proposing only one single most parsimonious tree (Figure 1 in main paper). We then relaunched the parsimony analysis of the matrices, this time asking for bootstrap support, using the following script:

log ds1_boot.log;

mxram 1000;

nstates NOGAPS;

taxname=;

p all_OG_1Copy_4spe_bpp_could_root.ss;

hold 1000;

rseed 0;

collapse 0;

ienum;

export - ds1_initial_intensive.best;

resample boot rep 1000 freq savetrees [mult=rep 1 hold 1];

export - ds1.intensive.boottrees;

log/;

quit;

PhySIC_IST offers the possibility to detect and correct outlier clades among the source trees. We can mainly set two parameters for this method: i) a confidence threshold b above which the clades of the source trees should be considered (in our case, this confidence value was inferred for each source tree by bootstrap from the alignment of the corresponding orthologous group); ii) a correction threshold c of strictness in correcting outlier clades form the source trees.

The analysis with the PhySIC_IST method was conducted for a large number of combinations of the STC (-c flag) and confidence (-b flag) parameters: from 0 to 1 varying by 0.1. The confidence support allows to account only for branches of the input trees having a support (e.g., bootstrap) above a given threshold. The STC parameter allows to change the behavior of the method from a purely optimization method (lower values of STC) to a strict consensus method (STC set to 1.0). More precisely, increasing STC (up to 100%) allows a smaller and smaller minority of trees to put a veto to proposed clades that contradict some of their triplets. Hence, ultimately, when set at 1.0, for any clade in the proposed supertree, all triplets induced by this clade must be present or induced by the input trees and, moreover, not contradicted by any of them. A typical command line to run PhySIC_IST was:

./PhySIC_IST-newMac.v1.1.0 -s ds2.tre -b $B -c $C -o physicist-b${B}-c${C}.tre -f newForest-b${B}-c${C}.tre > phys-b${B}-c${C}.out

where $B and $C are values for the confidence and STC parameters respectively, ds2.tre contains the gene trees of dataset #2, newForest-b${B}-c${C}.tre is the set of input trees modified to only keep branches with a threshold at least $B

The analysis with the superTriplets method was conducted as following:

java -jar -Xmx600m SuperTriplets_v1.1.jar rootedTress.tre superTripletSupportedClades.tre

and lead to the binary phylogeny. The reliability of each clade is based on the percentage of triplets of the input trees in agreement/disagreement with the clade (a triplet is a subtree connecting three given leaves. Any rooted input tree on n leaves can be equivalently represented by its set of O(n3) triplets). Note that superTriplets branch supports are more conservative than traditional bootstrap values. They mostly reflect the percentage of gene trees supporting the clade (independently of the number of considered gene trees).

The authors carried out a supermatrix analysis, both on Dataset #1 and #2 by concatenating all alignments of the orthologous groups composing a dataset. We thus obtained a supermatrix of 1,109,333 characters x 21 taxa containing only 34% of missing data for Dataset #1 and 541,931 characters x 21 taxa with 18% missing data for Dataset #2. We then estimated the most likely species tree according to each of these matrices separately, thanks to the IQ-TREE version 2 software. We used the edge-linked partition model to analyze the supermatrix, allowing each gene family to have its own evolutionary rate though all families shared the same branch lengths. We obtained branch support with the ultrafast bootstrap [12] by resampling partitions then sites within partitions.

We met a technical problem with the IQ-TREE method when analyzing Dataset #1, as distances between some taxa were too important (>3), which stopped the program at an intermediary inference step. To tackle the problem of studying too distant taxa, we temporarily removed the three outgroups (*Hepatocystis* sp., *Plasmodium falciparum* 3D7 and *P. gallinaceum* 8A) from the 2499 alignments, as *B. microti* was consistently found at the root of remaining taxa in the previous analyses (see above) and this could be used to root the obtained phylogeny. We discarded the alignments where less than 3 taxa remained. We thus obtained a data set (denoted #1’) of 2,381 alignments on 18 taxa.

Tree samples:

- MRP

From dataset #1

((BdunW,((CfelW,(TequW,((ToriS,(ToriF,ToriG)),(TannA,TparM)))),(BmicR,(Hpil2,(Pgal8,Pf3D7))))),(((BxinX,(BoviS,BbovF)),(BcabD,(BovaM,BbigB))),(Bmo1F,(Bdiv1,BdivR))));

With PhyML Bootstrap

(ToriF:0.01902491,ToriG:0.01481576,(ToriS:0.00000001,((TannA:0.00142389,TparM:0.00102794)100:0.07953690,(TequW:0.01282136,(CfelW:0.01651942,((BdunW:0.00609445,((BmoGF:0.00000001,(BdivR:0.00284104,BdivG:0.00689378)100:0.05757303)100:0.10101826,((BxinX:0.00215897,(BbovF:0.01250604,BoviS:0.00651609)100:0.06222349)100:0.09562510,(BcabD:0.01180556,(BbigB:0.00126298,BovaM:0.00108992)100:0.11136155)100:0.03087336)100:0.06988525)100:0.13588391)100:0.04311891,(BmicR:0.00442233,(Hpil2:0.00000001,(Pgal8:0.01592997,Pf3D7:0.01586667)100:0.04094167)100:0.13248621)100:0.08229858)100:0.07498309)100:0.03831524)100:0.11464958)100:0.10066643)100:0.03154716);

- PhySIC_IST with confident factor from dataset 2

(((Hpil2,(Pgal8,Pf3D7)55.4)100,(BmicR,((CfelW,(TequW,((TannA,TparM)96.7,(ToriS,(ToriG,ToriF)49.4)97)92.6)40.5)60.2,(BdunW,((Bmo1F,(Bdiv1,BdivR)83)99,((BcabD,(BovaM,BbigB)96.4)36.5,(BxinX,(BoviS,BbovF)73.5)75.8)65.4)95.4)50.7)80.2)100):0.0000000000;

- SuperTriplets with support

(((Pf3D7,Pgal8)55,Hpil2)100,((((((BdivR,Bdiv1)83,Bmo1F)99,((BovaM,BbigB)98,BcabD,((BoviS,BbovF)75,BxinX)82)73)97,BdunW)58,(TequW,((TparM,TannA)97,(ToriG,ToriF,ToriS)98)96,CfelW)74)83,BmicR)100);

- Super matrix with dataset #1

(ToriS:0.0706113988,(((((((((BoviS:0.2031976164,BbovF:0.3980474090):0.1147599795,BxinX:0.2494624721):0.1472209562,((BovaM:0.0780112597,BbigB:0.0857584247):0.2816494470,BcabD:0.3099744240):0.0454836912):0.1709173511,(Bmo1F:0.0180586829,(Bdiv1:0.0015619949,BdivR:0.0008510062):0.0103423142):0.4030948603):0.7437210735,BdunW:1.0050350068):0.1732543377,BmicR:2.5743476758):0.2769205668,CfelW:0.6911774661):0.1108373463,TequW:0.5273225306):0.6369920638,(TannA:0.0966684769,TparM:0.0926800225):0.3383463649):0.3648904977,(ToriF:0.0550107601,ToriG:0.0451767588):0.0212760259);

- Super matrix with dataset #2

(Pgal8:0.1064314407,(((((((TannA:0.0674276368,TparM:0.0629092051)100:0.2144271461,(ToriS:0.0448211435,(ToriF:0.0360463184,ToriG:0.0299960787)100:0.0143531254)100:0.2287617944)100:0.3864559684,TequW:0.3322368963)100:0.0698171744,CfelW:0.4328409590)100:0.1623446465,(((Bmo1F:0.0126225224,(Bdiv1:0.0011523844,BdivR:0.0008050027)100:0.0074767120)100:0.2490952175,(((BovaM:0.0519324115,BbigB:0.0557209125)100:0.1743791334,BcabD:0.1961292999)100:0.0311203505,(BxinX:0.1618722380,(BbovF:0.2494914854,BoviS:0.1323962495)100:0.0718957894)100:0.0929663209)100:0.1099934141)100:0.4411543291,BdunW:0.6223490019)100:0.1157227747)100:0.5131302484,BmicR:1.0152566998)100:1.8324573530,Hpil2:0.1903734173)100:0.0676408189,Pf3D7:0.1325278584);

***In vitro* drug efficacy.** The inhibitory effect of currently used anti-babesial drugs including atovaquone, clindamycin, azithromycin, quinine and an antifolate drug WR99210 on the intra-erythrocytic development of *B. MO1* parental isolates and clones B12 and F12 were tested and IC_50_ determination was performed using a previously reported protocol. Briefly, *B. MO1* parental isolate as well as two clones were cultured *in vitro* in human RBCs at 5% hematocrit (HC) in complete DMEM/F12 medium (Lonza, BE04-687F/U1). The parasite cultures (0.5% parasitemia, 5% HC in complete DMEM/F12 medium) were treated with decreasing concentrations of the compound of interest in a 96-well plate for 72 h. Following this, the parasitemia determination was performed using SYBR Green-I assay [1]. Briefly, 100 μl of the drug treated, or control parasite cultures were mixed with 100 μl of lysis buffer (0.008% saponin, 0.08% Triton-X-100, 20 mM Tris-HCl (pH = 7.5) and 5 mM EDTA) containing SYBR Green-I (0.01%) and incubated at 37°C for 1h in the dark. The fluorescence was measured at 480nm (excitation) and 540 nm (emission) by using a BioTek Synergy™ Mx Microplate Reader. The background fluorescence (uninfected RBCs in complete DMEM/F12 medium) was subtracted from each concentration and 50% inhibitory concentration (IC_50_) of the drug was determined by plotting sigmoidal dose-response curve fitting with drug concentration and percent parasite growth in the Graph Pad prism 9.4.1 from three independent experiments performed in triplicates. Data are shown as mean ± SD.

**DNA preparation for PacBio sequencing.** In vitro cultures of *B. MO1* clones B12 and F12 were initiated in human RBCs at 1% parasitemia, 5% HC (50 ml each) and cultured to attain 10% parasitemia. The cultures were harvested, and genomic DNA was isolated from both the clones using DNasy Blood and Tissue kit (Qiagen, Cat. No. 69506), The concentration determination and quality control was assessed using nanodrop and qubit, respectively. DNA integrity was determined using Blue Pippin pulse gel and following this, the DNA was used for library preparation using Pacific Biosciences SMRTbell Express template Prep Kit 2.0 (Cat. No. PN: 100-938-900) according to the manufacturer’s instructions. Loading concentration and proper stoichiometric measurements were determined using the Pacific Biosciences Smart Link software. Following this, the gDNA library was annealed to the Pacific Biosciences V5 primer for 1h at 20°C. The annealed library was then bound to polymerase using Pacific Biosciences Polymerase 2.2 for 1- 4h at 30°C and was loaded on to the Sequel II Instrument as an adaptive sequencing run. At least one smart cell was sequenced for each genomic DNA library with a movie time of 30h and a pre-extension of 2h. After the DNA library sequencing was complete, the loading metrics were evaluated by mean read length, polymerase read length, data yield and P1 values to ensure the sample ran as expected and data had met Yale's gold standards (polymerase read length between 50-60kb, data yield (HiFi) around 2-4 million reads of total 10-20Gb, and P1 between 60-70%).

**DNA preparation for Hi-C.** *In vitro* cultures of *B. MO1* clones B12 and F12 were initiated in human RBCs at 1% parasitemia, 5% HC (100 mL) and cultured to attain 10% parasitemia. The cultures were centrifuged, and the parasite pellets were cross-linked with 1.25% formaldehyde for 25 min at 37°C. Cross-linking reaction was quenched by the addition of 150mM (final concentration) glycine and incubation for 15 min at 37°C followed by a 15 min incubation at 4°C. This was followed by the lysis of parasite pellets by resuspension in lysis buffer (10 mM Tris-HCl, pH 8.0, 10 mM NaCl, 2 mM 4-(2-aminoethyl) benzenesulfonyl fluoride HCl (AEBSF), 0.25% Igepal CA-360 (v/v), and EDTA-free protease inhibitor cocktail (Roche)) and incubation for 30 min on ice. Nuclei were isolated after homogenization by 15 needle passages. *In situ* Hi-C protocol was conducted as described by Rao and colleagues [32]. Briefly, 0.5% sodium dodecyl sulfate (SDS) was used to permeabilize the nuclei. Subsequently, the DNA was digested using 100 units of Mbol (NEB), the ends of restriction fragments were filled using biotinylated nucleotides and ligated using T4 DNA ligase (NEB). After reversal of crosslinks, ligated DNA was purified and sheared to a length of ~300-500 bp using the Covaris ultrasonicator S220 (settings: 10% duty factor, 200 cycles per burst and a peak incident power of 140). Ligated fragments were pulled down using streptavidin beads (Invitrogen) and prepped for Illumina sequencing by subsequent end-repair, addition of A-overhangs and adapter ligation. Libraries were amplified for a total of 12 PCR cycles (45 sec at 98°C, 12 cycles of 15 sec at 98°C, 30 sec at 55°C, 30 sec at 62°C and a final extension of 5 min at 62°C) and sequenced with the NOVASeq platform (Illumina), generating 100 bp paired-end sequence reads at the UCSD core facility. Hi-C libraries were prepared in duplicate as previously described [6,20]and sequenced to a mean depth of ~98.4 million and 119 million reads for clones F12 and B12 respectively resulting in ~29.6 million and ~49.6 million valid interaction pairs contacts [21].

**RNA preparation for Illumina RNA-seq.** *B. MO1* clones B12 and F12 were cultured to a parasitemia of 8% at 5% HC (10mL culture volume per clone). Total RNA was isolated from clones B12 and F12 using five volumes of Trizol LS Reagent (Life Technologies, Carlsbad, CA, USA) and following manufacturer’s instructions. Total RNA was subjected to DNA-free DNA removal kit (ThermoFisher; AM1906) for removal of contaminating DNA. Following this, mRNA was purified from total RNA using NEBNext Poly(A) mRNA Magnetic Isolation Module (NEB, E7490S), and RNA-seq library was constructed using NEBNext Ultra II RNA-library preparation kit (NEB, E7770S) according to the manufacturer’s instructions. The RNA-libraries were amplified for 15 PCR cycles (45s at 98°C followed by 15 cycles of [15s at 98°C, 30s at 55°C, 30s at 62°C], 5 min 62°C). Next, the libraries were sequenced at 150 bp paired-end sequenced on the Illumina Novaseq platform (Illumina, San Diego, CA) at the UCSD and Yale core facility.

**Oxford Nanopore Sequencing.** DNA from *B. divergens* Rouen 87 and *Babesia* MO1 was not sheared and was used directly from purification for library construction. An ONT genomic DNA library was prepared by Ligation using the kit SQK-LSK109 following the vendor's protocol. A size-selection step was done at the last purification step after adapter ligation using Large Fragment Buffer (LFB) to wash AMpure XP beads, just before loading the library in the MinION R9.4.1 flow-cell. Base calling was performed with the Guppy software requesting High Accuracy Calling on a laptop with Graphic Processing Units (GPU’s).

**DNA preparation for Bionano optical map.** Exactly 3 ml packed frozen pellets of *B. MO1* in human RBCs were used to isolate ultra-high molecular weight (uHMW) genomic DNA for use in genomic optical mapping by Histogenetics (Ossining, NY) using the Bionano Prep™ Blood and Cell Culture DNA Isolation Kit (Bionano Genomics, cat No. 80004). Following this, DNA was quantified using Qubit™ dsDNA BR Assay Kit. A total of 0.75 ug of HMW DNA was then labeled using the Bionano Prep direct label and stain (DLS) method (Bionano Genomics, cat No. 80005) and loaded onto a flow cell to run on the Saphyr optical mapping system (Bionano Genomics). Approximately 1,177 Gb of data was generated per run. Raw optical mapping molecules in the form of BNX files were run through a preliminary bioinformatic pipeline that filtered out molecules less than 150 kb in size with and less than 9 motifs per molecule to generate a *de novo* assembly of the genome maps.

**Illumina sequencing.** Extracted DNA passed standard quantity, quality and purity assessments via determination of the 260/280nm for values of 1.7-2.0, and 260/230 absorbance ratios for values ≥ and 1% agarose gel electrophoresis to ensure that the gDNA is neither degraded nor displays RNA contamination. The library preparation started with 0.5ug of well quantified gDNA and underwent enzymatic fragmentation, end-repair and “A” base in a single reaction using Lotus DNA Library Prep kit (IDT, Part#10001074). The adapters with appropriate dual multiplexing indices, xGen UDI-UMI Adapters (IDT, Part #10005903), were ligated to the ends of the DNA fragments for hybridization to the flow-cell for cluster generation. Size of the final library construct was determined on Caliper LabChip GXsystem, and quantification was performed by qPCR SYBR Green reactions with a set of DNA standards using the Kapa Library Quantification Kit (KAPA Biosystems, Part#KK4854). For sequencing, the sample concentrations were normalized to 2nM and loaded onto Illumina NovaSeq 6000 S4 flow cells at a concentration that yields the requested number of passing filter data per lane. Samples were sequenced using 151 bp paired-end sequencing reads according to Illumina protocols.

**PacBio Iso-Seq library preparation and sequencing of *Babesia* MO1.** TRIzol reagent (Life Technologies, Carlsbad, CA, USA, No. 15596–026) was used to isolate total RNA from 100 ml *in vitro* culture of *B.* MO1 (15% parasitemia and 5% hematocrit) according to the manufacturer’s protocol. 1 µg of total RNA was used for the synthesis and amplification of cDNA using a combination of NEBNext Single Cell/Low Input cDNA Synthesis & Amplification module (Cat. No. E6421S), NEBNext High-Fidelity 2X PCR Master Mix (Cat. No. M0541S), Iso-Seq Express Oligo Kit (Cat. No. PN 101-737-500), and elution buffer (Cat. No. PN 101-633-500). SMRTbell libraries were constructed according to the Iso-Seq Express Template Protocol (Pacific Biosciences). Primer annealing and polymerase binding were performed following the SMRT Link v8.0 Sample Setup instructions and 90 pM of the SMRTbell templates were loaded for sequencing. One SMRT Cell 8M was used for each sample and sequencing was performed using the Sequel II system.

**Illumina RNA-Seq library preparation and sequencing of *B. divergens* Rouen 87.** Free merozoites and intraerythrocytic parasites were collected from two highly parasitized independent asynchronous *B*. *divergens* cultures, 75 ml each at parasitemias of 40% Total RNA from *B. divergens* free merozoites and intraerythrocytic parasites was prepared using Trizol LS Reagent (Life Technologies, Carlsbad, CA, USA, No. 15596–026) and chloroform extraction. Libraries were prepared using the Illumina Kit (Illumina) following the manufacturer’s protocol. High quality RNA samples from three biological replicates of free merozoites and from intraerythrocytic stages were used to prepare three independent libraries for each stage. The libraries were sequenced using the Illumina HiSeq platform with a paired-end configuration.

**PacBio HiFi sequencing.** Genomic DNA was isolated from 100 ml *in vitro* culture of *B.* MO1 (15% parasitemia and 5% hematocrit) using DNasy Blood and Tissue kit (Qiagen; Cat. No. 69506), and quality control along with concentration determination was performed by using nanodrop and qubit. DNA integrity was evaluated using Blue Pippin pulse gel and the DNA was then used for library preparation using Pacific Biosciences SMRTbell Express template Prep Kit 2.0 (Cat. No. PN: 100-938-900) according to the manufacturer’s instructions. The Pacific Biosciences Smart Link software was used to determine loading concentration and proper stoichiometric measurements. The gDNA library was then annealed to the Pacific Biosciences V5 primer for 1h at 20°C. The annealed library was then bound to polymerase using Pacific Biosciences Polymerase 2.2 for 1- 4h at 30°C and was loaded on to the Sequell II Instrument as an adaptive sequencing run. At least one smart cell was sequenced for each genomic DNA library with a movie time of 30h and a pre-extension of 2h. After the DNA library sequencing was complete, the loading metrics were evaluated by mean read length, polymerase read length, data yield and P1 values to ensure the sample ran as expected and data had met Yale's gold standards.

**Hi-C data processing.** Illumina reads were mapped using BWA MEM 0.7.17 [22] Contact maps were produced using HiC-Explorer v3.7.2 [21].

**Three-dimensional modeling.** Three-dimensional coordinate matrices were generated from the HiCexplorer output matrices using PASTIS [23]. The coordinate matrices were then converted to PDB format and visualized as 3D chromatin models in ChimeraX [24] and 10-kb bins containing telomeres and the approximate location of centromeres were highlighted.

**Pulse field gel electrophoresis (PFGE)**

Cultures of *B. divergens* MO1 and *B.* MO1 clones (B12, H1, F12, H6, A3 and F1), *B. divergens* Rouen 87 and *B. divergens* clones (H2, H6, C1, C7, A6 and H10) and the *B. divergens* clinical isolate from Spain were centrifuged at 1.300 x g for 5 min to yield pellets containing intact cells. Pellets, were embedded in 1% (w/v) SeaKem Gold Agarose (Lonza, Rockland, ME, USA) to an approximately concentration of 1x10^8^ infected RBCs/ml. The resultant agarose plugs were incubated in lysis solution (100mM EDTA, pH8.0, 0.2% sodium deoxycholate, 1% sodium lauryl sarcosine) supplemented with 1 mg/ml of proteinase K (Thermo Fisher Scientific, Vilnius, Lithuania) for 24 h at 50°C. Finally, plugs were washed 4 times for 30 min each in wash buffer (20 mM Tris, pH 8.0, 50 mM EDTA). Intact chromosomes were separated on a 0.8% Megabase Agarose gel (Bio-Rad Labs Inc., Hercules, CA, USA) in 1X TAE buffer chilled at 14°C for 48 h for *B. divergens* MO1 and *B. MO1* clones and 72 h for *B. divergens* Rouen 87, *B. divergens* clones and the *B. divergens* clinical isolate from Spain on a CHEF MapperTM XA pulsed field electrophoresis system (Bio-Rad). The switch time was 20 min-40 min-23 sec at 2V/cm with an include angle of 106⁰. The agarose gel was stained with GelRed (Biotium, Fremont, CA, USA) and visualized under ultraviolet transilluminator.

**Southern Blot Analysis**

Telomeric ends of *B. divergens* clinical isolate form Spain chromosomes were analyzed by Southern Blot using a nucleotide repeat sequence (CCCTGAACCCTAAA) of the telomeric ends of *Plasmodium berghei* chromosomes. The telomeric probe was labeled using the DIG Oligonucleotide Tailing Kit, 2nd Generation (Cat. No. 03353383910, Roche, Mannheim, Germany).

After PFGE and before transfer, DNA from agarose gels were depurinated (20 min in 0.25 M HCL), denatured (2 X 20 min in 0.5N NaOH; 1.5 M NaCl) and neutralized (2 X 20 min in 0.5 M Tris HCl, pH 7.5; 1.5 M NaCl). Southern blotting was done on nylon membrane, positively charged (Cat. No. 1417240, Roche) using 10X SSC and followed by UV crosslinking of transferred DNA.

A membrane was hybridized overnight at 26⁰C with the telomeric probe and washed twice in 2X SSC and 0.1% SDS for 5 min. Then, the membrane was washed twice in 0.5X SSC and 0.1% SDS at 26°C for 20 min.

Bound probe was detected with disodium-2-chloro-5(4 methoxyspiro (1,2-dioxetane-3.2´-[5-chloro]tricycle[3.3.1.1.3.7 55 ] decan)-4-yl)-1-phenyl phosphate (CDP-StarTM, Cat. No.12041677001, Roche) according to the manufacturer’s instructions. All membranes were visualized using an Amersham ImageQuant 800 58 system (GE Healthcare Bio-Science AB, Uppsala, Sweden.

**References**

[1] Singh P, Pal AC, Mamoun CB. An Alternative Culture Medium for Continuous In Vitro Propagation of the Human Pathogen Babesia duncani in Human Erythrocytes. Pathogens. 2022 May 20;11(5).

[2] Cheng H, Concepcion GT, Feng X, et al. Haplotype-resolved de novo assembly using phased assembly graphs with hifiasm. Nat Methods. 2021 Feb;18(2):170-175.

[3] Koren S, Walenz BP, Berlin K, et al. Canu: scalable and accurate long-read assembly via adaptive k-mer weighting and repeat separation. Genome Res. 2017 May;27(5):722-736.

[4] Gonzalez LM, Estrada K, Grande R, et al. Comparative and functional genomics of the protozoan parasite Babesia divergens highlighting the invasion and egress processes. PLoS Negl Trop Dis. 2019 Aug;13(8):e0007680.

[5] Walker BJ, Abeel T, Shea T, et al. Pilon: an integrated tool for comprehensive microbial variant detection and genome assembly improvement. PLoS One. 2014;9(11):e112963.

[6] Singh P, Lonardi S, Liang Q, et al. Babesia duncani multi-omics identifies virulence factors and drug targets. Nat Microbiol. 2023 May;8(5):845-59.

[7] Edgar RC. Muscle5: High-accuracy alignment ensembles enable unbiased assessments of sequence homology and phylogeny. Nat Commun. 2022 Nov 15;13(1):6968.

[8] Di Franco A, Poujol R, Baurain D, et al. Evaluating the usefulness of alignment filtering methods to reduce the impact of errors on evolutionary inferences. BMC Evol Biol. 2019 Jan 11;19(1):21.

[9] Ranwez V, Douzery EJP, Cambon C, et al. MACSE v2: Toolkit for the Alignment of Coding Sequences Accounting for Frameshifts and Stop Codons. Mol Biol Evol. 2018 Oct 1;35(10):2582-2584.

[10] Nguyen LT, Schmidt HA, von Haeseler A, et al. IQ-TREE: a fast and effective stochastic algorithm for estimating maximum-likelihood phylogenies. Mol Biol Evol. 2015 Jan;32(1):268-74.

[11] Chernomor O, von Haeseler A, Minh BQ. Terrace Aware Data Structure for Phylogenomic Inference from Supermatrices. Syst Biol. 2016 Nov;65(6):997-1008.

[12] Hoang DT, Chernomor O, von Haeseler A, et al. UFBoot2: Improving the Ultrafast Bootstrap Approximation. Mol Biol Evol. 2018 Feb 1;35(2):518-522.

[13] Kalyaanamoorthy S, Minh BQ, Wong TKF, et al. ModelFinder: fast model selection for accurate phylogenetic estimates. Nat Methods. 2017 Jun;14(6):587-589.

[14] Baum BR, Ragan MA. The MRP method. Phylogenetic supertrees: combining information to reveal the Tree of Life. 2004:17-34.

[15] Scornavacca C, Berry V, Lefort V, et al. PhySIC_IST: cleaning source trees to infer more informative supertrees. BMC Bioinformatics. 2008 Oct 4;9:413.

[16] Ranwez V, Criscuolo A, Douzery EJ. SuperTriplets: a triplet-based supertree approach to phylogenomics. Bioinformatics. 2010 Jun 15;26(12):i115-23.

[17] Gadagkar SR, Rosenberg MS, Kumar S. Inferring species phylogenies from multiple genes: concatenated sequence tree versus consensus gene tree. J Exp Zool B Mol Dev Evol. 2005 Jan 15;304(1):64-74.

[18] Seo TK, Kishino H, Thorne JL. Incorporating gene-specific variation when inferring and evaluating optimal evolutionary tree topologies from multilocus sequence data. Proc Natl Acad Sci U S A. 2005 Mar 22;102(12):4436-41.

[19] Lu J, Salzberg SL. SkewIT: The Skew Index Test for large-scale GC Skew analysis of bacterial genomes. PLoS Comput Biol. 2020 Dec;16(12):e1008439.

[20] Bunnik EM, Cook KB, Varoquaux N, et al. Changes in genome organization of parasite-specific gene families during the Plasmodium transmission stages. Nat Commun. 2018 May 15;9(1):1910.

[21] Ramirez F, Bhardwaj V, Arrigoni L, et al. High-resolution TADs reveal DNA sequences underlying genome organization in flies. Nat Commun. 2018 Jan 15;9(1):189.

[22] Li H. Aligning sequence reads, clone sequences and assembly contigs with BWA-MEM. arXiv preprint arXiv:13033997. 2013.

[23] Varoquaux N, Ay F, Noble WS, et al. A statistical approach for inferring the 3D structure of the genome. Bioinformatics. 2014 Jun 15;30(12):i26-33.

[24] Meng EC, Goddard TD, Pettersen EF, et al. UCSF ChimeraX: Tools for structure building and analysis. Protein Sci. 2023 Nov;32(11):e4792.

**Supplementary Figure Legends (Clean version)**

**Figure S1.** Growth of *B. divergens* Rouen 87 clones H2 and H6, and *B.* MO1 clones B12 and F12 in human RBCs in DMEM/F12 medium + 20% fetal bovine serum (FBS) or DMEM/F12 medium + 0.5% albumax over a course of 4 days. Two independent experiments were performed in triplicates.

**Figure S2**. Chromosomal organization of *Babesia* *divergens* clinical isolates from France and Spain by PFGE and subsequent Southern blot analyses using a *Plasmodium berghei* telomeric probe. **A**. PFGE (lines 1-5) and Southern-blot (lines 1*-5*) show the number and approximate sizes of chromosomes of *B. divergens* clinical isolates from France. **B**. PFGE (lane 6) and Southern-blot (line 6*) show the number and approximate sizes of chromosomes of the *B. divergens* clinical isolate from Spain. *Schizosaccharomyces cerevisiae* (Sc), *Hansenula wingei* and *Schizosaccharomyces pombe* (Sp) DNA chromosomes were used as DNA markers. The manufacture’s estimates of the sizes of chromosomes are indicated in Megabase pairs on the right and left of Panel A and on the left of panel B [13]. The Table shows epidemiologic and genomic features of the *B. divergens* clinical isolates. [33] [13].

**Figure S3A.** Visualization of the alignment of the *B.* MO1 clone F12 assembly against the Bionano optical map. The green lines represent the optical map molecules, the blue lines represent assembled contigs (1 is ChrI, 2 is Chr2, 3 is Chr3, while the others are unplaced contigs); vertical lines indicate matching positions during the restriction enzyme mapping.

**Figure S3B.** Visualization of the alignment of the *B.* MO1 clone B12 assembly against the Bionano optical map. The green lines represent the optical map molecules, the blue lines represent assembled contigs (1 is Chr I, 2 is Chr II, 3 is Chr III, while the others are unplaced contigs); vertical lines indicate matching positions during the restriction enzyme mapping.

**Figure S4.** Synteny analysis of *B.* MO1 clone B12 (blue), *B.* MO1 clone F12 (orange), and the parental *B.* MO1 (green); gray shaded areas indicated synteny; the length of insertions is annotated; “ITS” indicate the presence of interstitial telomeric sequence in the assembly.

**Figure S5A.** Dot-plot alignment between *B.* MO1 clone F12 and *B.* MO1 clone B12 assembly; the three largest blocks correspond to chromosomes I-III; the dot-plot includes unplaced contigs.

**Figure S5B.** Dot-plot alignment between *B.* MO1 clone F12 and the parental *B.* MO1; the three largest blocks correspond to chromosomes I-III; the dot-plot includes unplaced contigs.

**Figure S6.** **Phylogenomic analysis. A.** Species phylogeny proposed by Matrix Representation Parsimony (MRP) supertree phylogenomic approaches. Displayed clade support values are estimated by bootstrap on dataset #1. The tree obtained with dataset #2 was identical. All bootstraps were at 100% with dataset #2. *Hepatocystis sp.* (ex *Piliocolobus tephrosceles* 2019), *Plasmodium falciparum* 3D7 and *P. gallinaceum* 8A were taken as outgroup. **B.** Species phylogeny proposed by Super Triplets super tree phylogenomic approaches. The tree was obtained from dataset #2. Displayed clade are confidence value (from 0 to 100) computed by the method with respect to the input trees and then considering only the clades with confidence value above 50. *Hepatocystis sp.* (ex *Piliocolobus tephrosceles* 2019), *Plasmodium falciparum* 3D7 and *P. gallinaceum* 8A were taken as outgroup. **C.** Species phylogeny proposed by super matrix phylogenomic approaches. using Dataset #1’ (see supplementary method)**. D.** Species phylogeny proposed by super matrix phylogenomic approaches based on Dataset #2. All bootstraps were at 100%. *Hepatocystis sp.* (ex *Piliocolobus tephrosceles* 2019), *Plasmodium falciparum* 3D7 and *P. gallinaceum* 8A were taken as outgroup.

**Figure S7.** **Functional analysis of *Babesia* MO1 gene depending on patristic distances.** Patristic distances were calculated from the trees of dataset #1 for all *Babesia* sp. MO1- *B. divergens* isolates pairs. OUT trees support the position of *Babesia* sp. MO1 as a new species. MIX trees places Babesia sp. MO1 between the two *B. divergens* isolates. **A**. Cumulative distribution of patristic distances among OUT and MIX trees. The X-axis is defined as –log10(patistitic distance). Higher distances are on the left part of the graph. Threshold values (vertical dashed lines) between High, medium, and Low set of genes were the lower and upper quartile of the value that were below 4. Genes with values higher than 4 were considered as non-significant (NS), which means too close to *B. divergens* genes to support any phylogenetic inference. All genes from MIX trees were considered as NS. **B.** GO term enrichment among the four sets of genes. The hypergeometric law was used to evaluate the p-value. GO terms were selected when more than two genes match the term in the subset and p-value was below 0.125. GO terms were ordered from top to bottom by descendant value of the median of patristic distance of all genes matching the terms in a subset. The color intensity is according to the p-value, red being the most significant.

**Figure S8.** Hi-C contact map of *B.* MO1 clone F12; the panels at the bottom are the contact maps for individual chromosomes; green circles/squares indicate the putative location of the centromeres.

**Figure S9.** Hi-C contact map of *B.* MO1 clone B12; the panels at the bottom are the contact maps for individual chromosomes.

**Figure S10.** Hi-C contact map of *B.* divergens Rouen 87; the panels at the bottom are the contact maps for individual chromosomes.

**Figure S11. In vitro efficacy of current antibabesial compounds against *B.* MO1 and *B. divergens* Rouen 87. A-F.** Potency and IC_50_ determination of Atovaquone (ATV), Azithromycin (AZT), Quinine [46], Clindamycin (CLN), WR99210 (WR), and Pyrimethamine (PYM) against *B. divergens* Rouen 87 clones H2 and H6, and *B.* MO1 clones B12 and F12. Data presented as mean ± SD of three independent experiments performed in biological triplicates.

**Figure S12**. Sequence alignment of DHFR-TS from different *Babesia* and *Plasmodium* species.
