# Supplementary material for: Insights into the evolution, virulence and speciation of Babesia MO1 and Babesia divergens through multiomics analyses
Source: Emerg Microbes Infect. 2024 Aug 15;13(1):2386136. doi: 10.1080/22221751.2024.2386136 (PMC11370697; doi:10.1080/22221751.2024.2386136)
Supplement: Supplementary figures 1 to 11.pdf [file TEMI_A_2386136_SM4000.pdf]

Figure S1

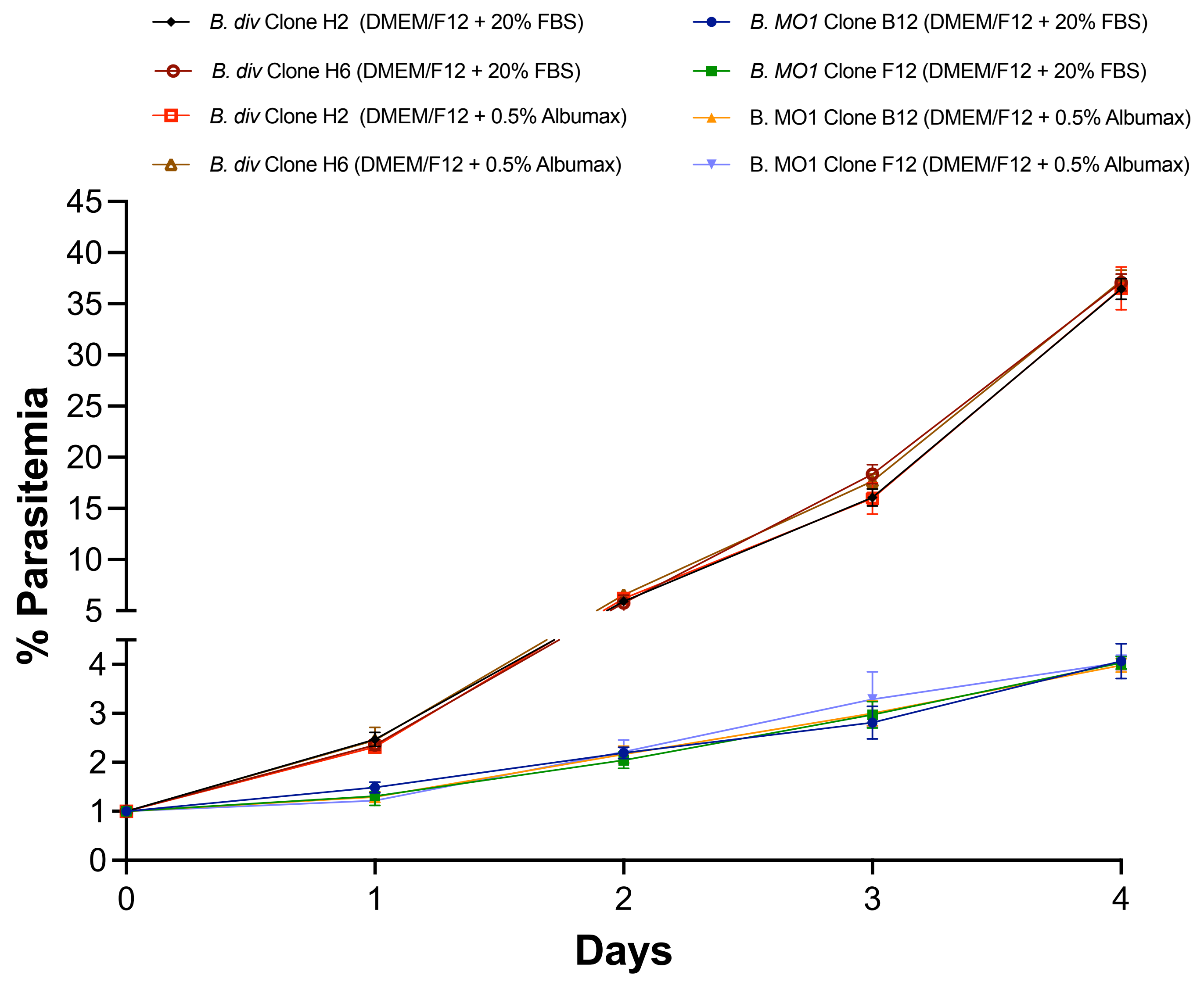

B. div Clone H2 (DMEM/F12 + 20% FBS)
B. div Clone H6 (DMEM/F12 + 20% FBS)
B. div Clone H2 (DMEM/F12 + 0.5% Albumax)
B. div Clone H6 (DMEM/F12 + 0.5% Albumax)
B. MO1 Clone B12 (DMEM/F12 + 20% FBS)
B. MO1 Clone F12 (DMEM/F12 + 20% FBS)
B. MO1 Clone B12 (DMEM/F12 + 0.5% Albumax)
B. MO1 Clone F12 (DMEM/F12 + 0.5% Albumax)
% Parasitemia
45
40
35
30
25
20
15
10
5
4
3
2
1
0
0
1
2
3
4
Days

Figure S2

A

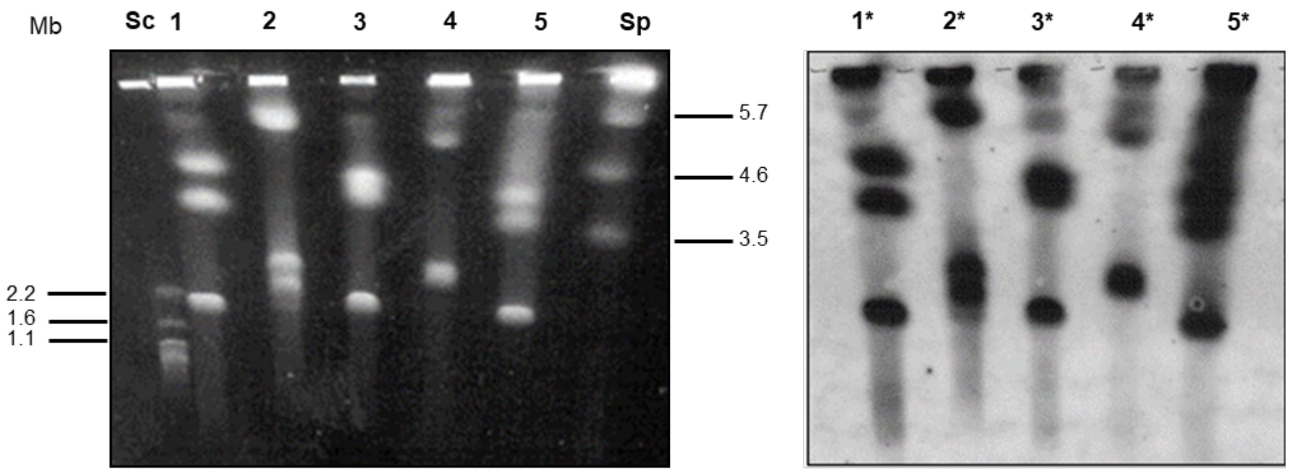

B

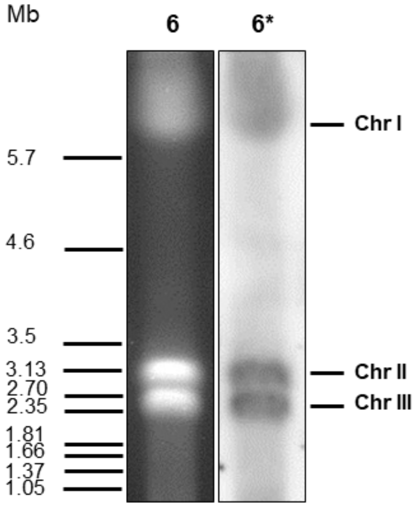

SAMPLE	1	2	3	4	5	6
HOST	Bovine	Bovine	Human	Bovine	Bovine	Human
ISOLATE ORIGIN	5008B Manche (Fr)	6303E Puy de Dome (Fr)	Rouen87 Seine-Maritime (Fr)	Y5 Ireland	W8843 Scotland	Asturias Spain
Chr I (Mb)	4.8	5.7	4.7	5.0	4.6	5.7
Chr II (Mb)	4.5	2.8	4.1	2.9	3.7	3.13
Chr III (Mb)	2.2	2.3	2.2	2.9	2.2	2.7
GENOME SIZE	10.5	10.8	11.0	10.8	10.5	11.55

Figure S3 A

B. MO1 clone F12 assembly vs. optical map

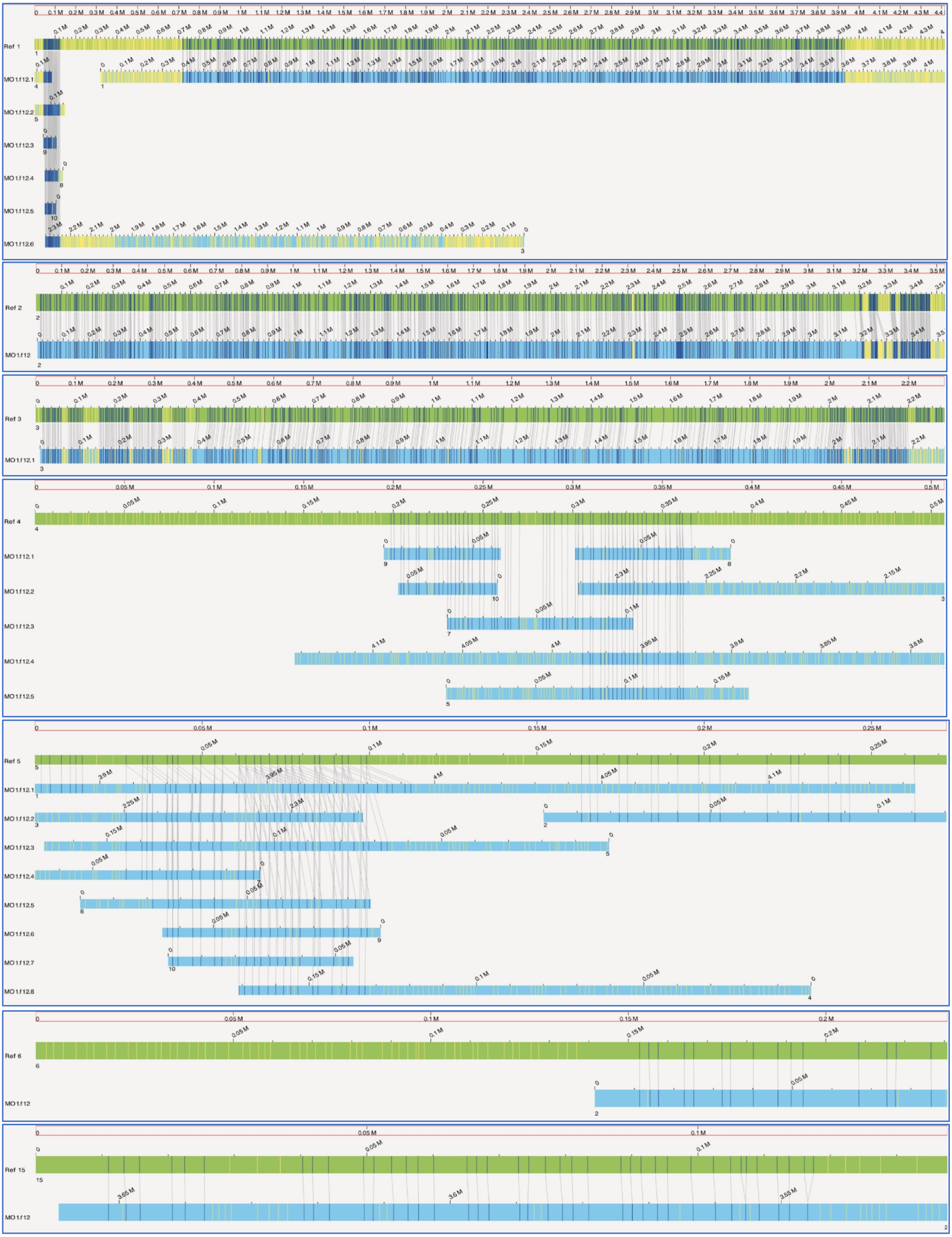

Figure S3 B

B. MO1 clone B12 assembly vs. optical map

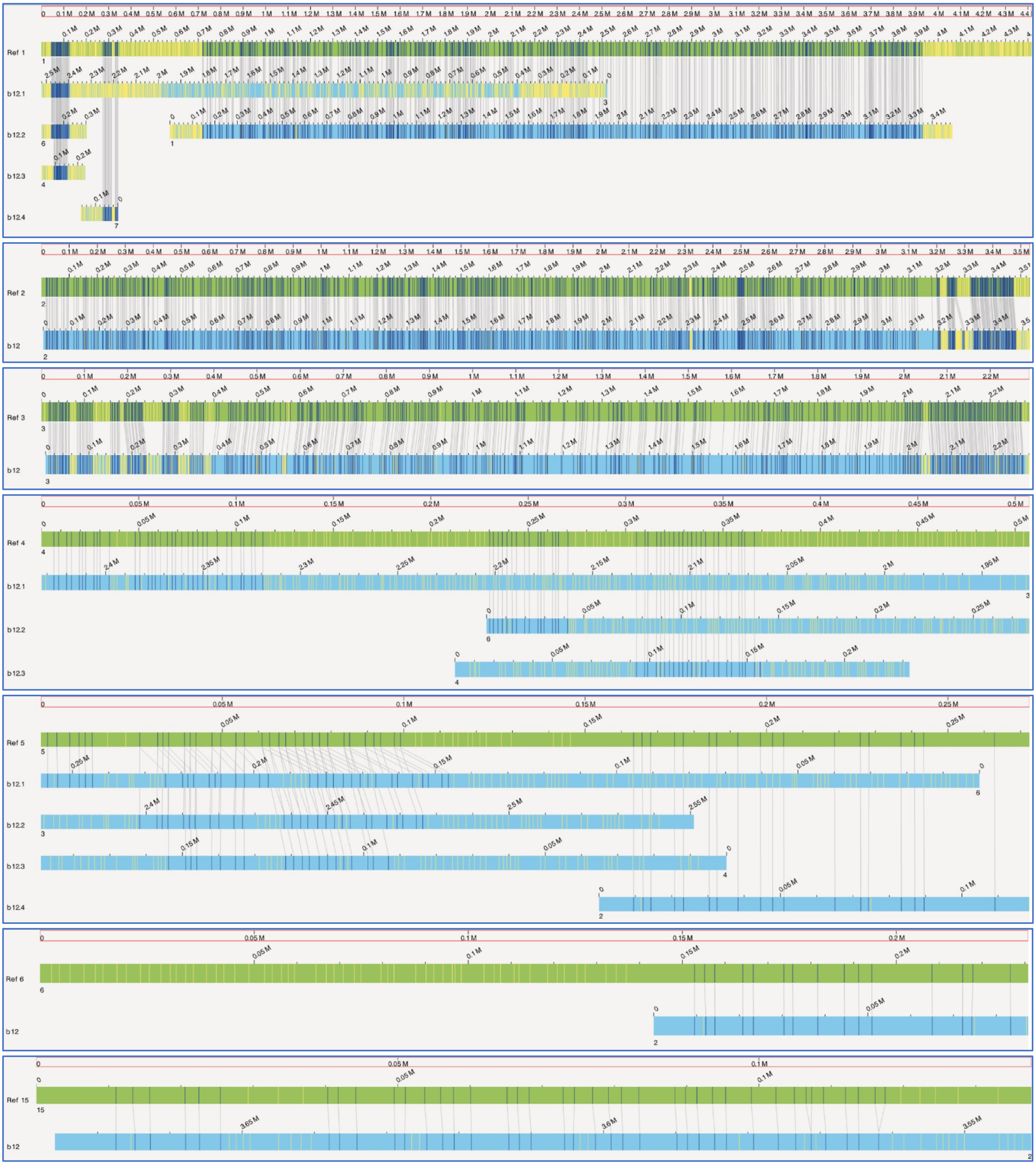

Figure S4

Synteny plot (clone F12, clone B12, parental *B.* MO1)

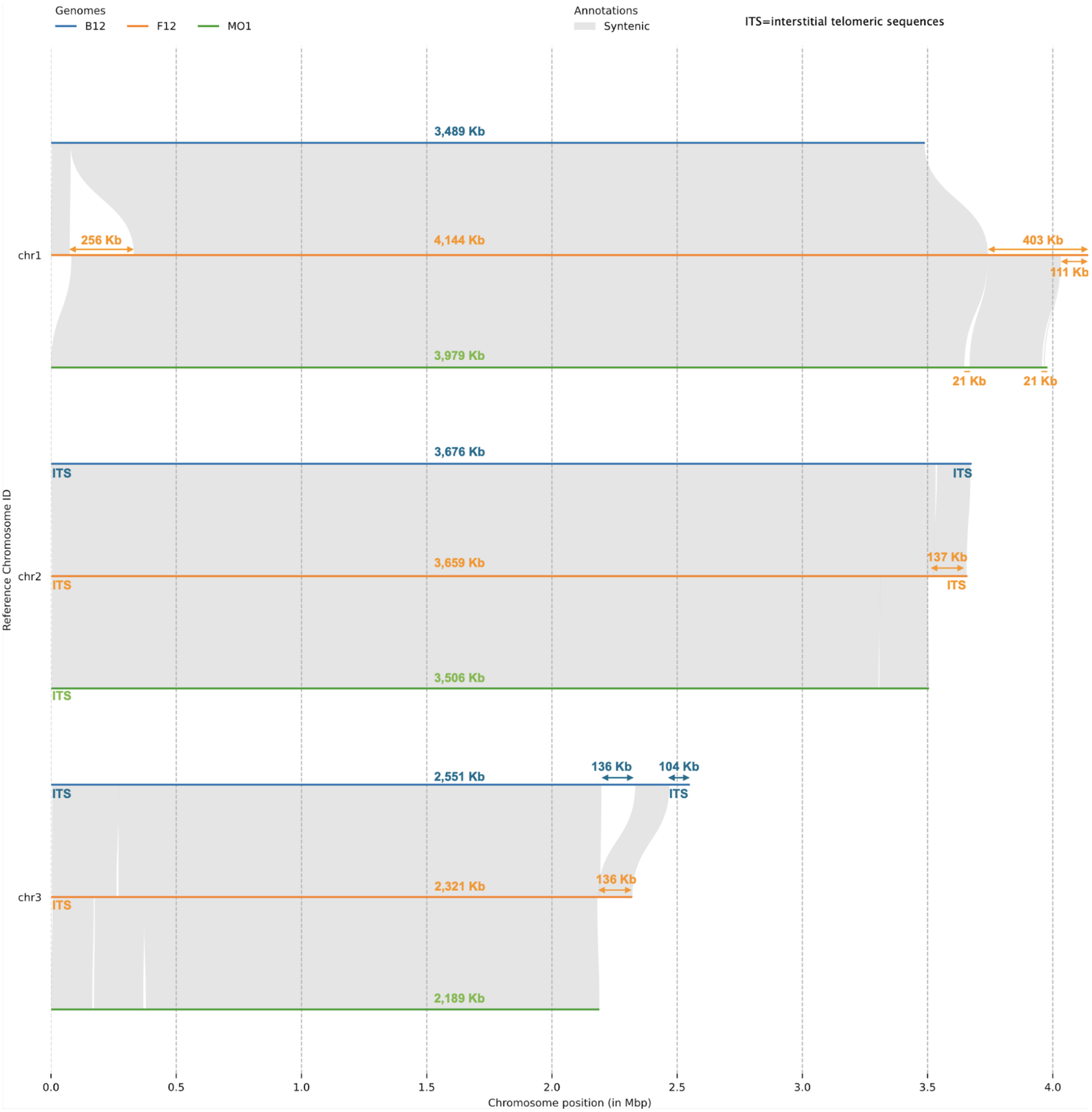

Figure S5 A

B. **MO1 clone F12 vs clone B12**

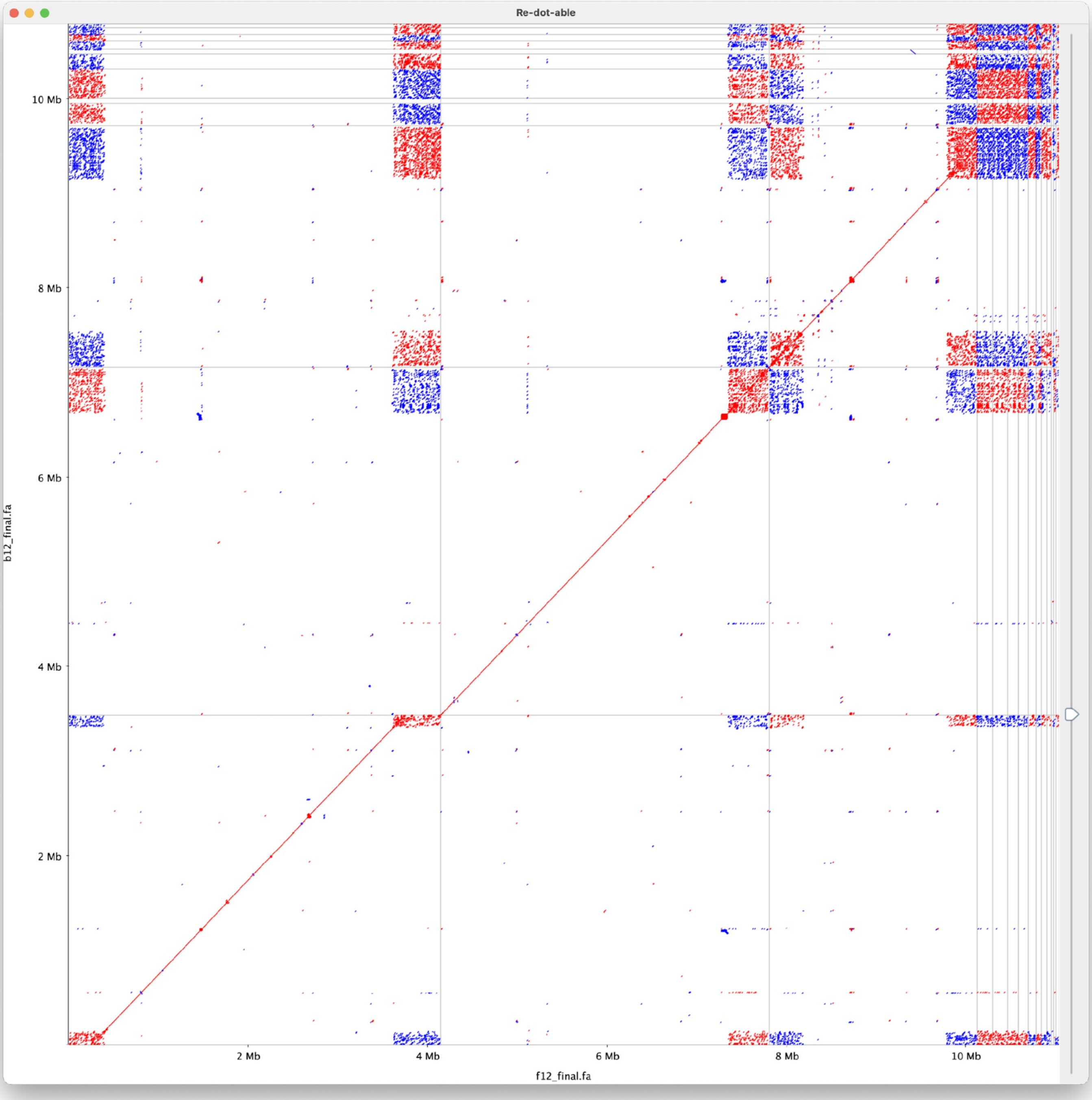

Figure S5 B

***B.* MO1 clone F12 vs parental *B.* MO1**

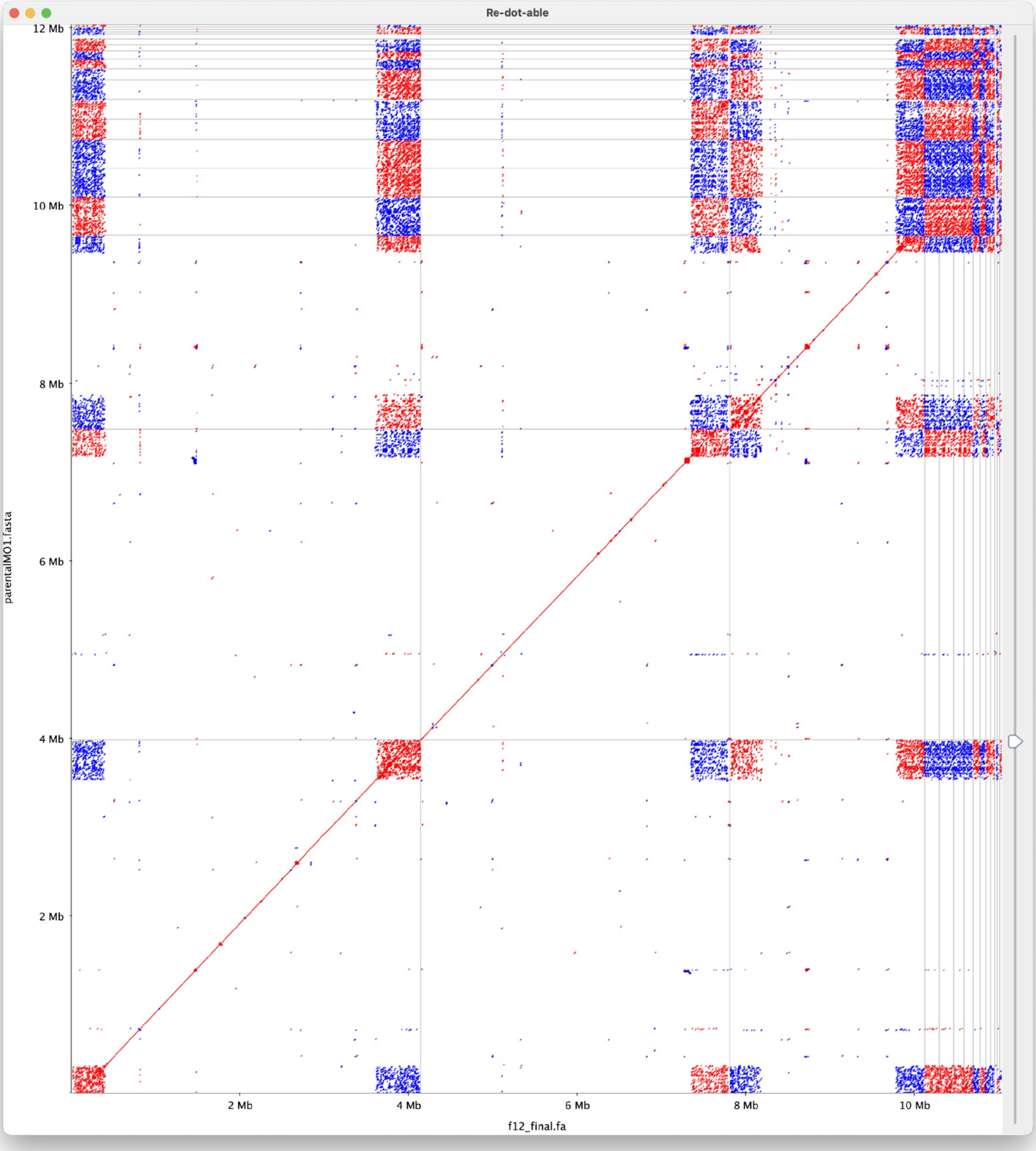

Figure S6

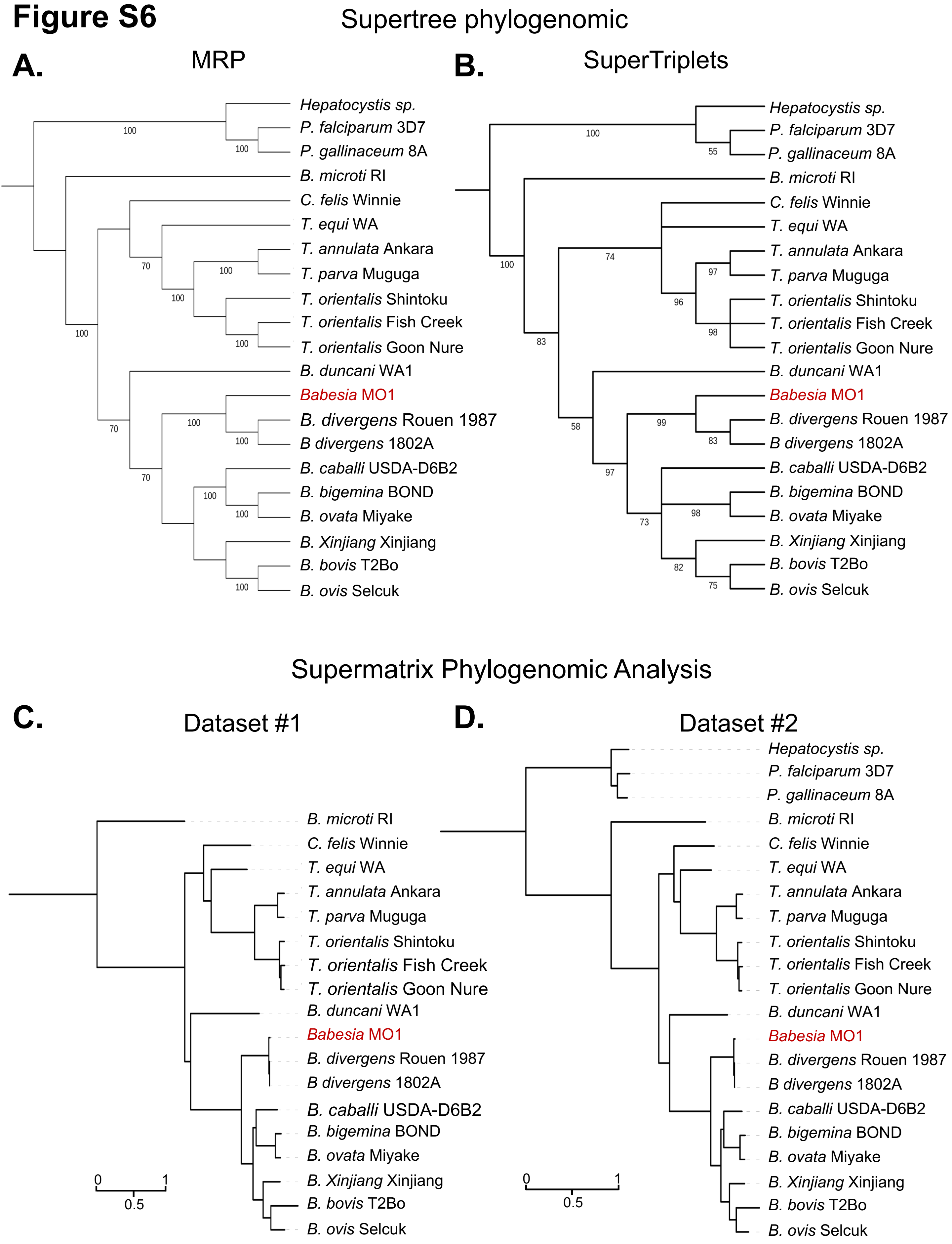
Supertree phylogenomic
A.
MRP
Hepatocystis sp.
P. falciparum 3D7
P. gallinaceum 8A
B. microti RI
C. felis Winnie
T. equi WA
T. annulata Ankara
T. parva Muguga
T. orientalis Shintoku
T. orientalis Fish Creek
T. orientalis Goon Nure
B. duncani WA1
Babesia MO1
B. divergens Rouen 1987
B divergens 1802A
B. caballi USDA-D6B2
B. bigemina BOND
B. ovata Miyake
B. Xinjiang Xinjiang
B. bovis T2Bo
B. ovis Selcuk
100
100
70
100
100
100
100
100
70
100
100
70
100
100
100
B.
SuperTriplets
Hepatocystis sp.
P. falciparum 3D7
P. gallinaceum 8A
B. microti RI
C. felis Winnie
T. equi WA
T. annulata Ankara
T. parva Muguga
T. orientalis Shintoku
T. orientalis Fish Creek
T. orientalis Goon Nure
B. duncani WA1
Babesia MO1
B. divergens Rouen 1987
B divergens 1802A
B. caballi USDA-D6B2
B. bigemina BOND
B. ovata Miyake
B. Xinjiang Xinjiang
B. bovis T2Bo
B. ovis Selcuk
100
55
100
74
97
96
98
83
58
99
83
97
98
73
82
75
Supermatrix Phylogenomic Analysis
C.
Dataset #1
B. microti RI
C. felis Winnie
T. equi WA
T. annulata Ankara
T. parva Muguga
T. orientalis Shintoku
T. orientalis Fish Creek
T. orientalis Goon Nure
B. duncani WA1
Babesia MO1
B. divergens Rouen 1987
B divergens 1802A
B. caballi USDA-D6B2
B. bigemina BOND
B. ovata Miyake
B. Xinjiang Xinjiang
B. bovis T2Bo
B. ovis Selcuk
0
1
0.5
D.
Dataset #2
Hepatocystis sp.
P. falciparum 3D7
P. gallinaceum 8A
B. microti RI
C. felis Winnie
T. equi WA
T. annulata Ankara
T. parva Muguga
T. orientalis Shintoku
T. orientalis Fish Creek
T. orientalis Goon Nure
B. duncani WA1
Babesia MO1
B. divergens Rouen 1987
B divergens 1802A
B. caballi USDA-D6B2
B. bigemina BOND
B. ovata Miyake
B. Xinjiang Xinjiang
B. bovis T2Bo
B. ovis Selcuk
0
1
0.5

Figure S7

A.

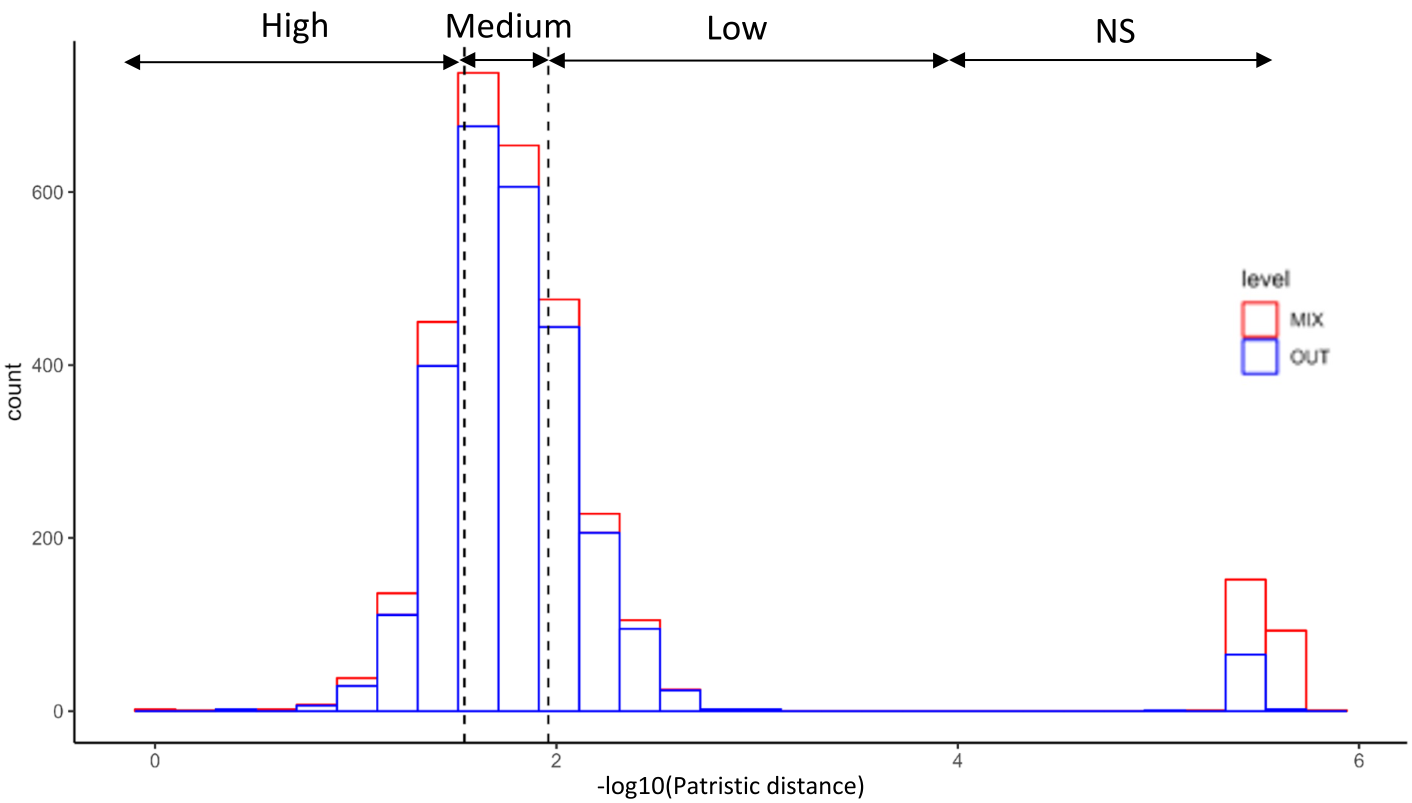
High
Medium
Low
NS
600
400
200
0
count
level
MIX
OUT
0
2
4
6
-log10(Patristic distance)

B.

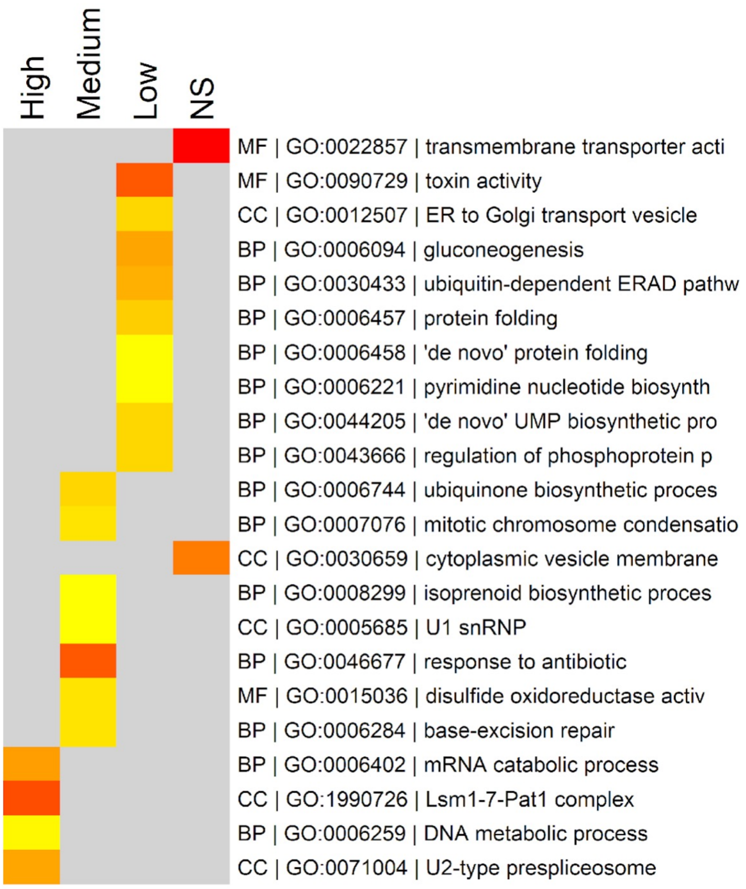
High
Medium
Low
NS
MF | GO:0022857 | transmembrane transporter acti
MF | GO:0090729 | toxin activity
CC | GO:0012507 | ER to Golgi transport vesicle
BP | GO:0006094 | gluconeogenesis
BP | GO:0030433 | ubiquitin-dependent ERAD pathw
BP | GO:0006457 | protein folding
BP | GO:0006458 | 'de novo' protein folding
BP | GO:0006221 | pyrimidine nucleotide biosynth
BP | GO:0044205 | 'de novo' UMP biosynthetic pro
BP | GO:0043666 | regulation of phosphoprotein p
BP | GO:0006744 | ubiquinone biosynthetic proces
BP | GO:0007076 | mitotic chromosome condensatio
CC | GO:0030659 | cytoplasmic vesicle membrane
BP | GO:0008299 | isoprenoid biosynthetic proces
CC | GO:0005685 | U1 snRNP
BP | GO:0046677 | response to antibiotic
MF | GO:0015036 | disulfide oxidoreductase activ
BP | GO:0006284 | base-excision repair
BP | GO:0006402 | mRNA catabolic process
CC | GO:1990726 | Lsm1-7-Pat1 complex
BP | GO:0006259 | DNA metabolic process
CC | GO:0071004 | U2-type prespliceosome

Figure S8

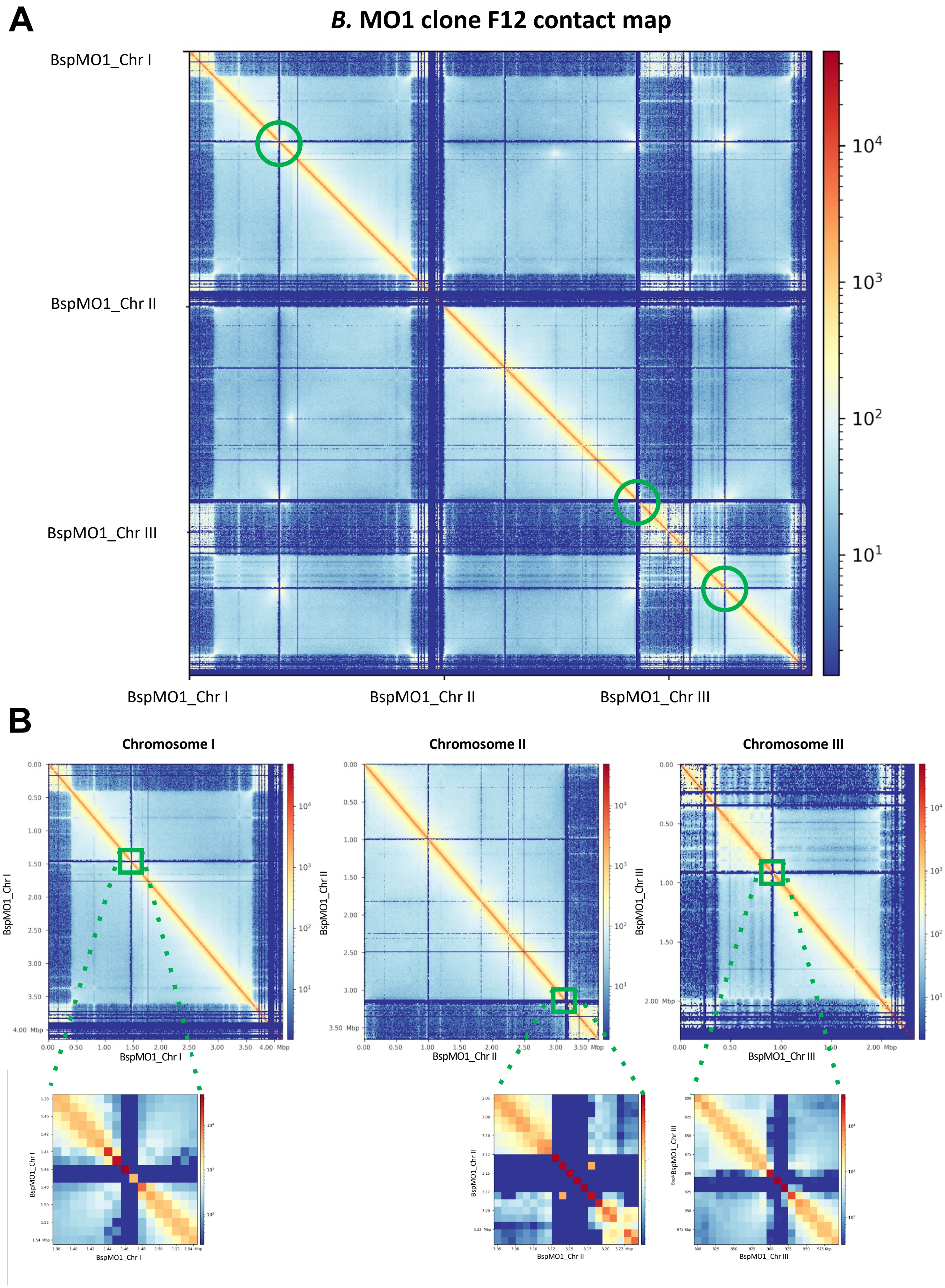
A
B. MO1 clone F12 contact map
BspMO1_Chr I
BspMO1_Chr II
BspMO1_Chr III
B
Chromosome I
Chromosome II
Chromosome III
BspMO1_Chr I
BspMO1_Chr II
BspMO1_Chr III

Figure S9

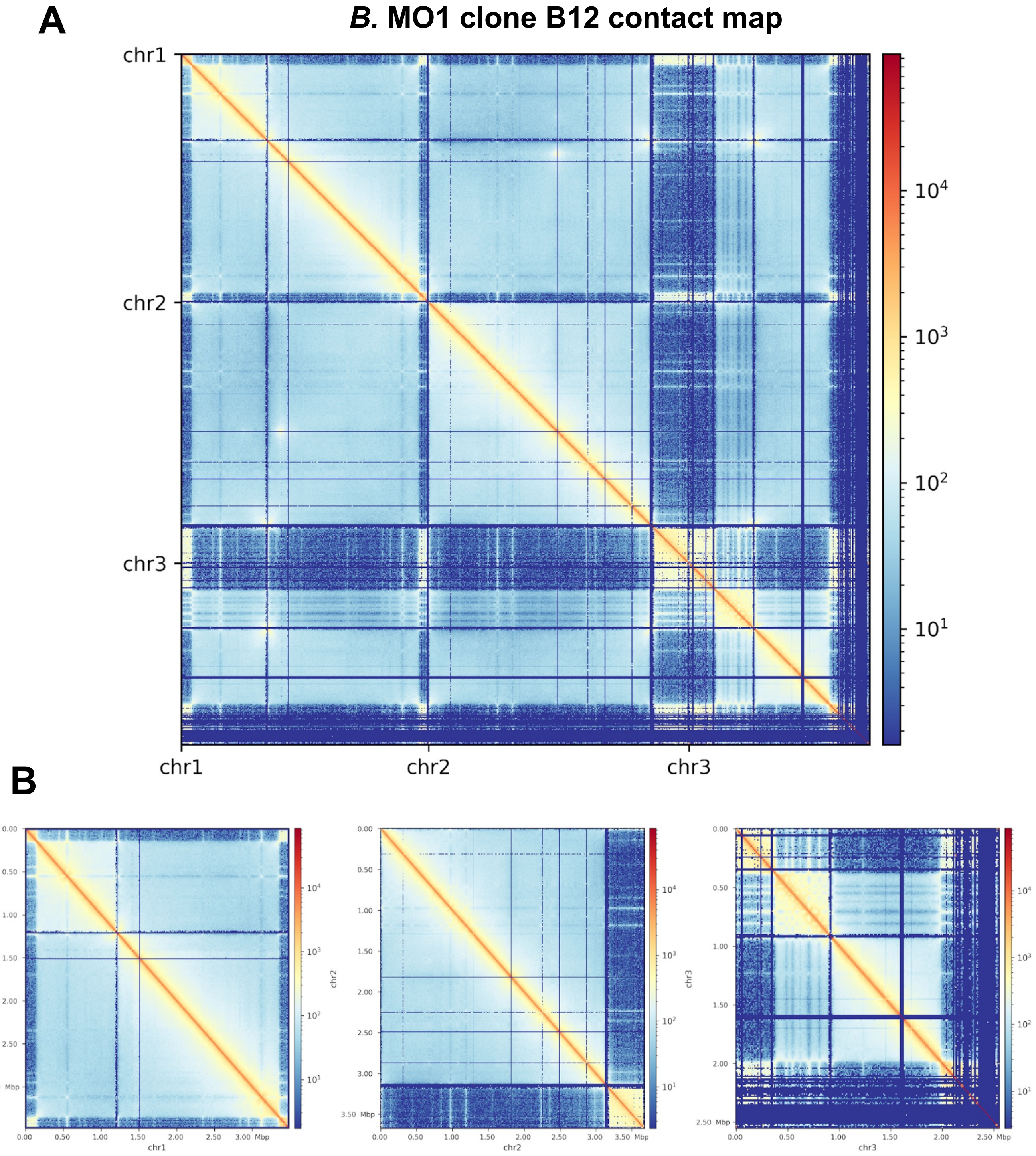
A
B. MO1 clone B12 contact map
chr1
chr2
chr3
10^4
10^3
10^2
10^1
B
chr1
chr2
chr3
Mbp

Figure S10

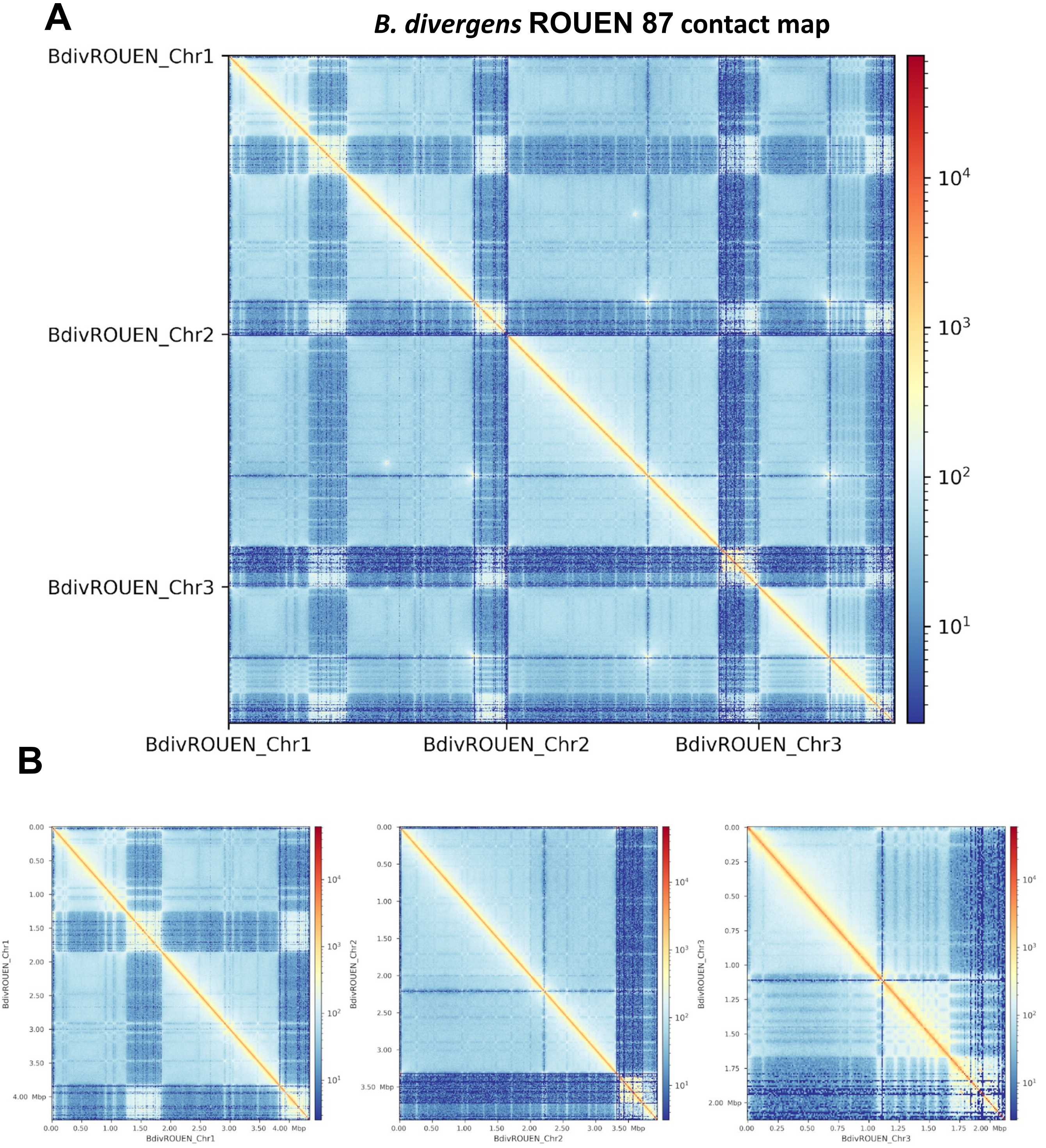
A
B. divergens ROUEN 87 contact map
BdivROUEN_Chr1
BdivROUEN_Chr2
BdivROUEN_Chr3
10^4
10^3
10^2
10^1
B
0.00 0.50 1.00 1.50 2.00 2.50 3.00 3.50 4.00 Mbp
BdivROUEN_Chr1
0.00 0.50 1.00 1.50 2.00 2.50 3.00 3.50 Mbp
BdivROUEN_Chr2
0.00 0.25 0.50 0.75 1.00 1.25 1.50 1.75 2.00 Mbp
BdivROUEN_Chr3

Figure S11

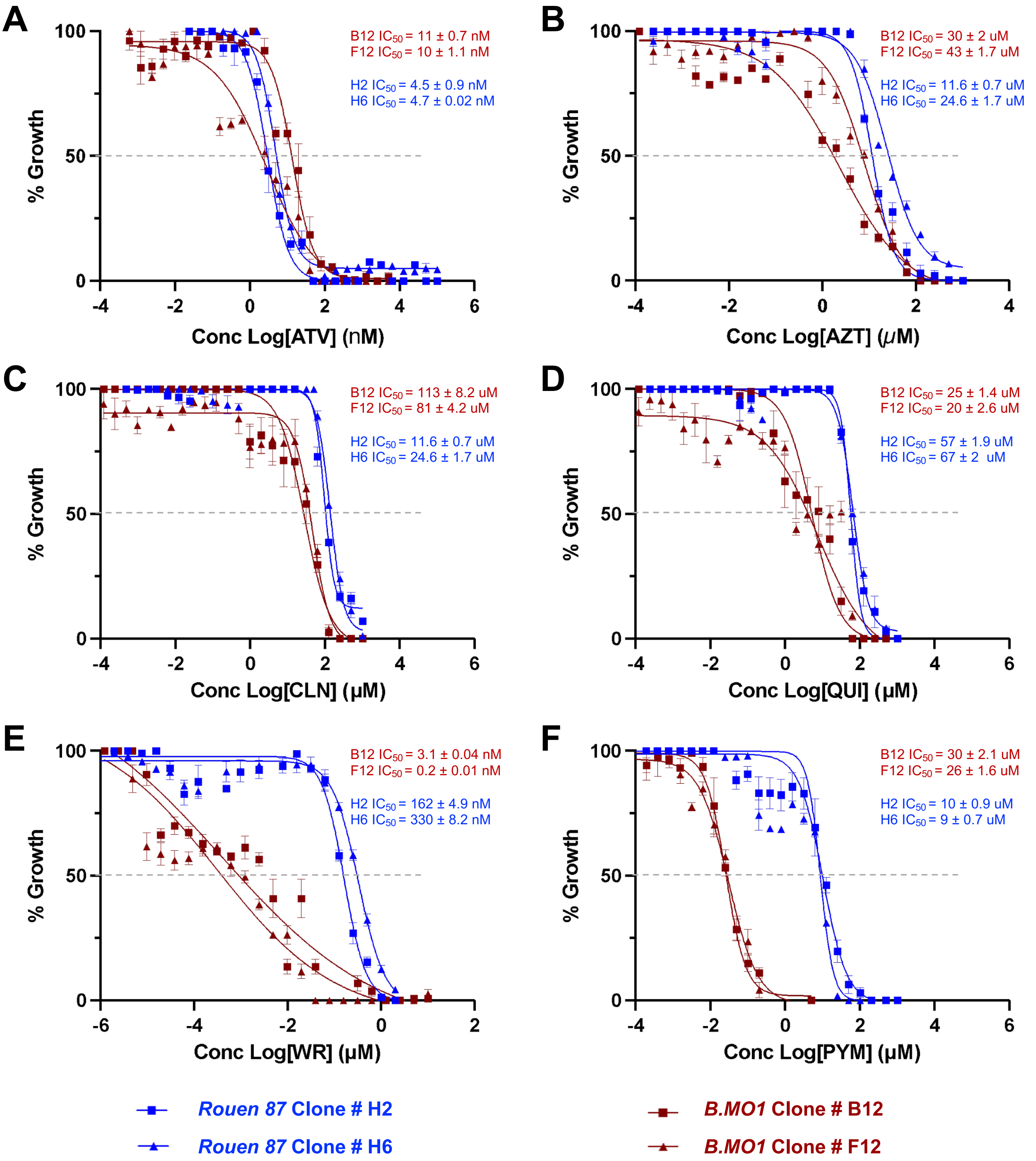

A
B12 IC50 = 11 ± 0.7 nM
F12 IC50 = 10 ± 1.1 nM
H2 IC50 = 4.5 ± 0.9 nM
H6 IC50 = 4.7 ± 0.02 nM
% Growth
Conc Log[ATV] (nM)
B
B12 IC50 = 30 ± 2 uM
F12 IC50 = 43 ± 1.7 uM
H2 IC50 = 11.6 ± 0.7 uM
H6 IC50 = 24.6 ± 1.7 uM
% Growth
Conc Log[AZT] (μM)
C
B12 IC50 = 113 ± 8.2 uM
F12 IC50 = 81 ± 4.2 uM
H2 IC50 = 11.6 ± 0.7 uM
H6 IC50 = 24.6 ± 1.7 uM
% Growth
Conc Log[CLN] (μM)
D
B12 IC50 = 25 ± 1.4 uM
F12 IC50 = 20 ± 2.6 uM
H2 IC50 = 57 ± 1.9 uM
H6 IC50 = 67 ± 2 uM
% Growth
Conc Log[QUI] (μM)
E
B12 IC50 = 3.1 ± 0.04 nM
F12 IC50 = 0.2 ± 0.01 nM
H2 IC50 = 162 ± 4.9 nM
H6 IC50 = 330 ± 8.2 nM
% Growth
Conc Log[WR] (μM)
F
B12 IC50 = 30 ± 2.1 uM
F12 IC50 = 26 ± 1.6 uM
H2 IC50 = 10 ± 0.9 uM
H6 IC50 = 9 ± 0.7 uM
% Growth
Conc Log[PYM] (μM)
Rouen 87 Clone # H2
Rouen 87 Clone # H6
B.MO1 Clone # B12
B.MO1 Clone # F12

Figure S12

DHFR-TS

```
Babesia MO1            1   ---MVANYEGCGGLEIYVATAWN     RAIGFKNDIPWPHIREDFRFLARGTSYVDPEVKAK     NPDLMNVVVMGRKT   69
B.divergens Rouen 87   1   ---MVANYEGCGGLEIFVATAWN     RAIGFKNDIPWPHIREDFRFLARGTSYVDPEVKAK     NPDLMNVVIMGRKT   69
P.falciparum 3D7       1   mMeQVCDVFDIYAICACCKVESK[14]RGLGNKGVLPWKCNSLDMKYFCAVTTYVNESKYEK[22]SKKLQNVVVMGRTS  108
P.falciparum HB3       1   mMeQVCDVFDIYAICACCKVESK[14]RGLGNKGVLPWKCNSLDMKYFCAVTTYVNESKYEK[22]SKKLQNVVVMGRTN  108
B.duncani WA1          1   -MdLSTKYEGFSPIVMFVATDVK     GGIGFEGKIPWPHIPMDFSFFFRGTCYVEPEILNR     YPEIQNVVIFGRKT   71
B.microti              1   -M-------GMYKVCSIYASTPN     GGIGNEGKLPWKTLPRDLKHLQDITTAYGPD----     -HSVQNVVIMGRKT   59
Babesia bovis          1   ---MSNSYEGCGDLTIFVAVALN     KVIGHKNQIPWPHITHDFRFLRNGTTYIPPEVLSK     NPDIQNVVIFGRKT   69

Babesia MO1            70   YESIPASSRPLKNRINVVLSRNVK--EI-PGCLVFPSLTEAIRHVRSSVPHYKIFCLGGGEVYREVMENDLCDRIYLTRL  146
B.divergens Rouen 87   70   YESIPESSRPLKNRINVVLSRNVK--DI-PGCLVFPSLTEAIRHVRSSVPHHKIFCLGGGEVYREVMENDLCDRIYLTRL  146
P.falciparum 3D7       109  WESIPKKFKPLSNRINVILSRTLKkeDFdEDVYIINKVEDLIVLL-GKLNYYKCFIIGGSVVYQEFLEKKLIKKIYFTRI  187
P.falciparum HB3       109  WESIPKKFKPLSNRINVILSRTLKkeDFdEDVYIINKVEDLIVLL-GKLNYYKCFIIGGSVVYQEFLEKKLIKKIYFTRI  187
B.duncani WA1          72   YESIPANVFPLKKRHNVIISRTLT--HV-PGASVFNNLDDALRWVNESKRFHKTIIMGGVEIYKLALETGLVEKIYLTRI  148
B.microti              60   YISIPKSSRPLKDRINIVLSSSVS--DFgDGVIAAKSMQDAFDKL-EKMKFNKIFIIGGSSVYKEAYDLGIVEKVYVTRV  136
Babesia bovis          70   YESIPKASLPLKNRINVILSRTVK--EV-PGCLVYEDLSTAIRDLRANVPHNKIFILGGSFLYKEVLDNGLCDKIYLTRL  146

Babesia MO1            147  TEEYEGDVFFPEIPD-TFQITGISKTFSTDYVTFDFVVYEKVG     AIK-EKRPPTFDELLLT     GGELTVPTPKYV  216
B.divergens Rouen 87   147  TEEYEGDVFFPEIPD-TFQITGISKTFSTDYVTFDFVVYEKTG     AIK-EKRPPTFDELLLT     GGELTVPTPKYV  216
P.falciparum 3D7       188  NSTYECDVFFPEINEnEYQIISVSDVYTSNNTTLDFIIYKKTN[30]MKKLTEFYKNVDKYKIN[24]KNKNSIHPNDFQ  313
P.falciparum HB3       188  NSTYECDVFFPEINEnEYQIISVSDVYTSNNTTLDFIIYKKTN[30]MKKLTEFYKNVDKYKIN[24]KNKNSIHPNDFQ  313
B.duncani WA1          149  GNEFKADRFFPDITK-DFEIVGISQTFGTDFTTFDFVIYEKKG     LNQLDHVTKSFDEMLLT     GKPLKTVAPLYK  219
B.microti              137  NKELPADTFVTSVPP-IFEIVGISRTFSYNDIPFDFIIYMLKD     SRATSNVCVDIDDYLLT     EHEIRDFKYQFK  207
Babesia bovis          147  NKEYPGDTYFPDIPD-TFEITAISPTFSTDFVSYDFVIYERKD     CKT-VFPDPPFDQLLMT     GTDISVPKPKYV  216

Babesia MO1            217  ACPSVKVRYHQEFQYLDIIADVLSTGTLKPNRTGVDGISKFGYQMHFDLSQSFPLLTTKKVALRSIIEELLWFIRGSTNG  296
B.divergens Rouen 87   217  ACPSVKVRYHQEFQYLDIIADVLSTGTLKPNRTGVDGISKFGYQMHFDLSQSFPLLTTKKVALRSIIEELLWFIRGSTNG  296
P.falciparum 3D7       314  IYNSLKYKYHPEYQYLNIIYDIMMNGNKQSDRTGVGVLSKFGYIMKFDLSQYFPLLTTKKLFLRGIIEELLWFIRGETNG  393
P.falciparum HB3       314  IYNSLKYKYHPEYQYLNIIYDIMMNGNKQSDRTGVGVLSKFGYIMKFDLSQYFPLLTTKKLFLRGIIEELLWFIRGETNG  393
B.duncani WA1          220  ACPNIRIRYHQEFQYLDICADVLSTGTLKENRTGIDALSKFGYQMRFDLSQSFPLLTTKKVFLRGITEELIWFINGSTNG  299
B.microti              208  ALPNVTIRKHEEIQYLDIIADILSSGSENDDRTGVGVLSKFGYKMEFNLDESFPLLTTKKVFFKGIVEELLWFIKGDTSG  287
Babesia bovis          217  ACPGVRIRNHEEFQYLDILADVLSHGVLKPNRTGTDAYSKFGYQMRFDLSRSFPLLTTKKVALRSIIEELLWFIKGSTNG  296

Babesia MO1            297  NELLDKNVRIWELNGARSFLDNLGFTDREEHDLGPVYGFQWRHFGAKYLDMHADYSGQGIDQLKVVINKIKTNPNDRRLI  376
B.divergens Rouen 87   297  NELLDKNVRIWELNGARSFLDNLGFTDREEHDLGPVYGFQWRHFGAKYLDMHADYSGQGIDQLKVVINKIKTNPNDRRLI  376
P.falciparum 3D7       394  NTLLNKNVRIWEANGTREFLDNRKLFHREVNDLGPIYGFQWRHFGAEYTNMYDNYENKGVDQLKNIINLIKNDPTSRRIL  473
P.falciparum HB3       394  NTLLNKNVRIWEANGTREFLDNRKLFHREVNDLGPIYGFQWRHFGAEYTNMYDNYENKGVDQLKNIINLIKNDPTSRRIL  473
B.duncani WA1          300  NDLLKKNVRIWELNGKRSFLDNLGFYNREEHDLGPVYGFQWRHFGATYTDMHADYKGQGIDQLVNVINSIKNDPNSRRLI  379
B.microti              288  KILLEKGVRIWEKNGTREFLDSVGLNERKEHDLGPIYGFQWRHFGAEYKDCDTNYTGQGIDQLMEAIDKIKNDPNSRRII  367
Babesia bovis          297  NDLLAKNVRIWELNGRRDFLDKNGFTDREEHDLGPIYGFQWRHFGAEYLDMHADYTGKGIDQLAEIINRIKTNPNDRRLI  376

Babesia MO1            377  ICSWNVADLSKMALPPCHCLYQFYVRDGKLSCMLHQRSCDLGLGVPFNIASYAILTAMIAQVCGLKLGEFVHNLADAHVY  456
B.divergens Rouen 87   377  ICSWNVADLSKMALPPCHCLYQFYVRDGKLSCMLHQRSCDLGLGVPFNIASYAILTAMIAQVCGLKLGEFVHNLADAHVY  456
P.falciparum 3D7       474  LCAWNVKDLDQMALPPCHILCQFYVFDGKLSCIMYQRSCDLGLGVPFNIASYSIFTHMIAQVCNLQPAQFIHVLGNAHVY  553
P.falciparum HB3       474  LCAWNVKDLDQMALPPCHILCQFYVFDGKLSCIMYQRSCDLGLGVPFNIASYSIFTHMIAQVCNLQPAQFIHVLGNAHVY  553
B.duncani WA1          380  VCSWNVSDVPKMALPPCHLLFQFYVAQGKLSCMLHQRSCDLGLGVPFNIASYSILTAMIAQVCNLQLGEFVHNLADAHIY  459
B.microti              368  VCSWNVNDLSKMALPPCHCLFQFYVSQGRLSCIMYQRSADVGLGVPFNIASYSLLTLMIAQVCALRPGKFVHVLGNAHIY  447
Babesia bovis          377  VCSWNVSDLKKMALPPCHCFFQFYVSDNKLSCMMHQRSCDLGLGVPFNIASYSILTAMVAQVCGLGLGEFVHNLADAHIY  456

Babesia MO1            457  IDHVDAMKLQMSRIPYPFPQLKLNPAITNIEDFTIDDIIVQNYVSHPPIKMAMSA  511
B.divergens Rouen 87   457  VDHVDAMKLQMSRIPYPFPQLKLNPAIKNIEDFTIDDIIVQNYVSHPPIKMAMSA  511
P.falciparum 3D7       554  NNHIDSLKIQLNRIPYPFPTLKLNPDIKNIEDFTISDFTIQNYVHHEKISMDMAA  608
P.falciparum HB3       554  NNHIDSLKIQLNRIPYPFPTLKLNPDIKNIEDFTISDFTIQNYVHHEKISMDMAA  608
B.duncani WA1          460  VNHIDALKEQLTRVPYPFPLLKLNKDITDICDFKLEDVKVEGYSCHPTIKMEMAA  514
B.microti              448  KTHITALTKQIQRIPYPFPILKLNAQVKRIEDFVPSDINLLCYTCHPTIKMDMAA  502
Babesia bovis          457  VDHVDAVTTQIARIPHPFPRLRLNPDIRNIEDFTIDDIVVEDYVSHPPIPMAMSA  511
```
